# Oscillatory Activity in the Medial Prefrontal Cortex and Nucleus Accumbens Correlates with Impulsivity and Reward Outcome

**DOI:** 10.1371/journal.pone.0111300

**Published:** 2014-10-21

**Authors:** Nicholas A. Donnelly, Tahl Holtzman, P. Dylan Rich, Alejo J. Nevado-Holgado, Anushka B. P. Fernando, Gert Van Dijck, Tobias Holzhammer, Oliver Paul, Patrick Ruther, Ole Paulsen, Trevor W. Robbins, Jeffrey W. Dalley

**Affiliations:** 1 Behavioural and Clinical Neuroscience Institute, University of Cambridge, Cambridge, United Kingdom; 2 Department of Psychology, University of Cambridge, Cambridge, United Kingdom; 3 Howard Hughes Medical Institute, Janelia Farm Research Campus, Ashburn, Virginia, United States of America; 4 Department of Experimental Psychology, University of Oxford, Oxford, United Kingdom; 5 Department of Microsystems Engineering, Microsystem Materials Laboratory, University of Freiburg, Freiburg, Germany; 6 Department of Physiology, Development and Neuroscience, University of Cambridge, Cambridge, United Kingdom; 7 Department of Psychiatry, University of Cambridge, Cambridge, United Kingdom; Federal University of Rio Grande do Norte, Brazil

## Abstract

Actions expressed prematurely without regard for their consequences are considered impulsive. Such behaviour is governed by a network of brain regions including the prefrontal cortex (PFC) and nucleus accumbens (NAcb) and is prevalent in disorders including attention deficit hyperactivity disorder (ADHD) and drug addiction. However, little is known of the relationship between neural activity in these regions and specific forms of impulsive behaviour. In the present study we investigated local field potential (LFP) oscillations in distinct sub-regions of the PFC and NAcb on a 5-choice serial reaction time task (5-CSRTT), which measures sustained, spatially-divided visual attention and action restraint. The main findings show that power in gamma frequency (50–60 Hz) LFP oscillations transiently increases in the PFC and NAcb during both the anticipation of a cue signalling the spatial location of a nose-poke response and again following correct responses. Gamma oscillations were coupled to low-frequency delta oscillations in both regions; this coupling strengthened specifically when an error response was made. Theta (7–9 Hz) LFP power in the PFC and NAcb increased during the waiting period and was also related to response outcome. Additionally, both gamma and theta power were significantly affected by upcoming premature responses as rats waited for the visual cue to respond. In a subgroup of rats showing persistently high levels of impulsivity we found that impulsivity was associated with increased error signals following a nose-poke response, as well as reduced signals of previous trial outcome during the waiting period. Collectively, these in-vivo neurophysiological findings further implicate the PFC and NAcb in anticipatory impulsive responses and provide evidence that abnormalities in the encoding of rewarding outcomes may underlie trait-like impulsive behaviour.

## Introduction

Impulsivity is a multi-faceted behaviour encompassing actions that are inappropriately timed and executed without due consideration of their consequences. It is a major component of several neuropsychiatric disorders, including attention deficit hyperactivity disorder (ADHD), drug addiction and Parkinson's disease [1]–[3], and can be divided into several sub-categories including delay aversion, action cancellation, reflection impulsivity and action restraint [2], [4]–[6]. High levels of impulsivity in humans [7]–[9] and rodents [10], [11] can both precede the development of drug addiction and contribute to drug relapse [12], [13]. The neural substrates of several forms of impulsivity converge on the prefrontal cortex (PFC) and topographically-organised inputs from this region to the nucleus accumbens core (NAcbC) and shell (NAcbSh) [2], [6], [14]–[17], with lesions of distinct sub-regions of the PFC [18]–[20] and NAcb [21], [22] selectively modulating impulsive responding in rats.

Previous studies indicate that single unit activity [23]–[26] and power in local field potential oscillations (LFPs) in PFC [27] encode waiting, future rewards and previous trial outcomes on various appetitive tasks, while single unit activity and gamma-frequency (50–80 Hz) LFPs in the NAcb have been linked to reward and response vigour [28]–[32]. However, to date, there has been little research on neural circuit activity within the PFC and NAcb in the context of either impulsive behaviours on a single-trial basis or individual differences in trait-like impulsivity. Therefore, in the present study, we investigated the neurophysiological substrates of impulsivity in rats performing the 5-choice serial reaction time task (5-CSRTT). The 5-CSRTT is a widely used paradigm to assess sustained visual attention and action restraint, requiring rats to wait for the presentation of visual cues instructing which action to select [33].

Rats that show persistently high levels of impulsivity on the 5-CSRTT (i.e., repeated failures in action restraint, or waiting impulsivity) show abnormal dopamine (DA) and γ-amino-butyric acid (GABA) function in the NAcb [10], [34]–[37] and manipulations of the DA innervation of the NAcb affect impulsivity on this task [38]–[40]. DA also modulates gamma and theta-frequency LFPs and single unit activity in NAcb [28], [41], [42], including the GABAergic interneurons in NAcb which are implicated in the generation of gamma oscillations [43]. Therefore we investigated the relationship of LFP oscillations in the gamma and theta frequency bands, and oscillatory phase-amplitude coupling, to 5-CSRTT behaviour through simultaneous LFP recordings in the PFC and NAcb. We found that LFP oscillations correlated with key events associated with 5-CSRTT performance, and that LFP activity was significantly different before impulsive acts and correlated with trait-like impulsivity in a group of highly impulsive animals.

## Materials and Methods

### Animals

Male outbred Lister-hooded rats (n = 17; Charles River, Margate, UK) were used in this study. Animals were group housed 4 per cage at 20°C under diurnal conditions (12-h light, 12-h dark), food deprived at 85% of free-feeding weight, and given access to water *ad libitum*. Rats were singly housed following electrode implantation, which occurred at 6–8 months of age. Behavioural testing was conducted at the same time each day during the animal's dark phase. All experimental procedures were carried out in accordance with the United Kingdom Animals (Scientific Procedures) Act of 1986 and were approved by ethical review at the University of Cambridge. Throughout the experiments, all efforts were made to minimise suffering.

### Behavioural apparatus and training

**Figure 1:**
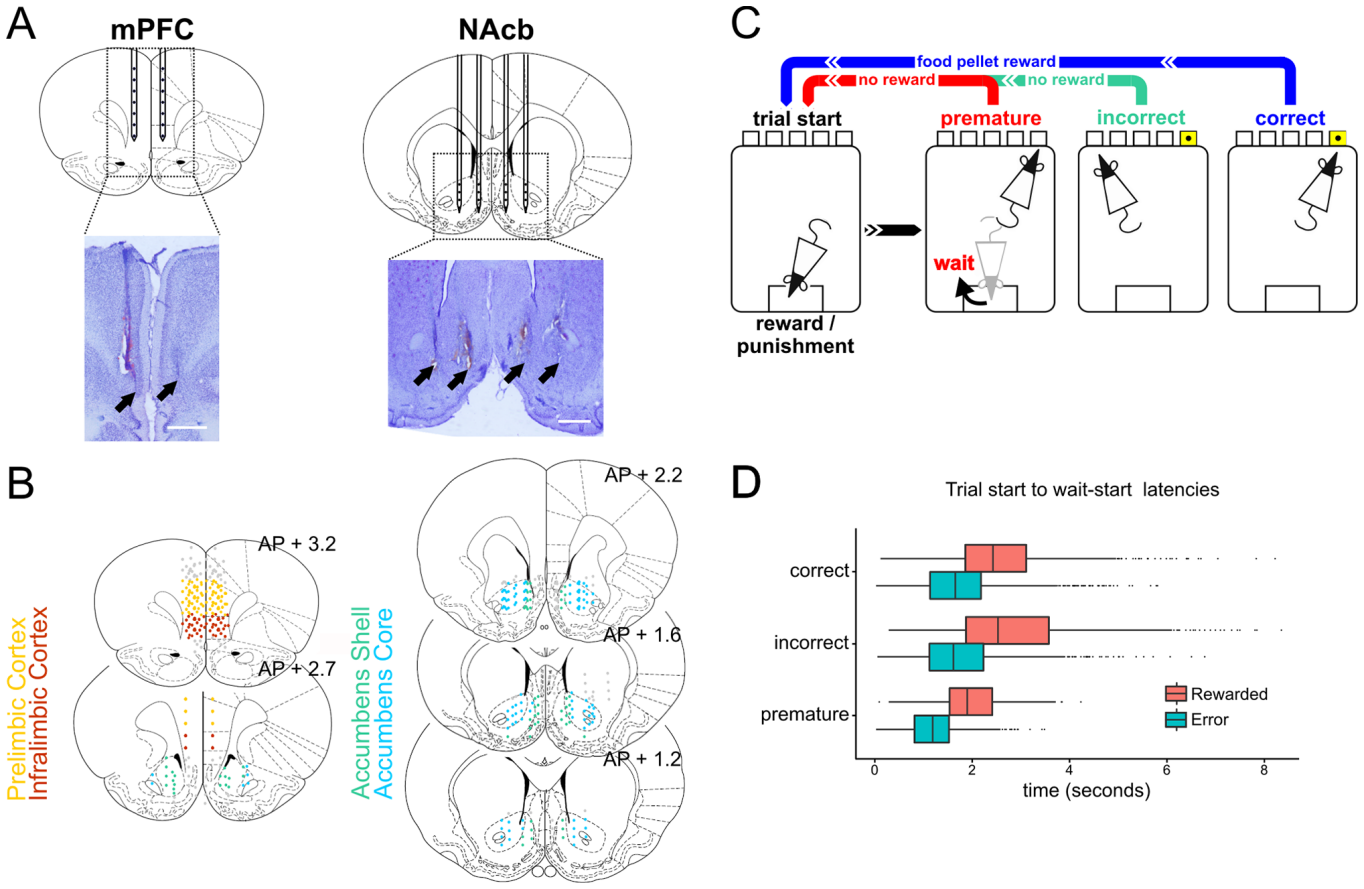
Electrode placements and behavioural data. A) Representative histology of silicon probe placement in the medial prefrontal cortex and nucleus accumbens. B) Reconstructed placements of all electrode contacts in prelimbic and infralimbic prefrontal cortex and nucleus accumbens core and shell. C) Scheme of 5-Choice Serial Reaction Time Task (5-CSRTT). Trials start with a nose-poke in the food magazine. After a 5 second delay a 0.5 second light stimulus is presented pseudorandomly in one of 5 nose-poke ports. A response to the illuminated hole within 5 seconds is rewarded with a food pellet. Responses during the waiting period, to the wrong hole, or the absence of a response within 5 seconds of stimulus presentation are punished with a 5 second lights-off timeout. D) Distribution of behavioural latencies for rats to move from entering the food magazine, starting a new trial, to leaving the magazine to start waiting, split by the outcome of the previous trial (either ending in a correct response, and being rewarded, or ending in an incorrect or premature error response). Boxes show the range from 1^st^ to 3^rd^ quartile of responses, black lines show the median, and whiskers extend to the furthest value from the hinge within 1.5 times the inter-quartile range. Values outside this range are represented as black dots.

Rats were trained on the 5-CSRTT (Figure 1C, [33], [44]) using the method described by Bari et al. (2008). All testing was carried out in operant chambers (Med Associates, VT, USA), controlled by a PC running Whisker software [45]. In this task rats initiate a trial by nose-poking at the food magazine, triggering a delay of 5 seconds (this delay has also been termed an inter-trial interval [33]). At the end of this delay a 0.5 second light stimulus is presented in one of 5 nose-poke holes. A nose-poke in the illuminated hole within 5 seconds (the limited hold) is rewarded with a food pellet (Noyes Dustless 45 mg Pellet, Sandown Scientific, Middlesex, UK) dispensed in the food magazine. The commission of a correct response produces rapid discrete cues that reward is available (i.e. the sound of the food dispenser and the illumination of a light in the food magazine). The next trial is started as soon as the rat returns to the food magazine to collect the food pellet. Nose pokes made before the presentation of the stimulus (premature, or impulsive responses), nose pokes to a non-illuminated hole after stimulus presentation (incorrect responses), or failure to make any nose-poke response during the limited hold (omissions) are punished with a 5 second time-out period, during which time the chamber houselight was extinguished and no food pellets are available. The end of the timeout is signalled by the houselight and a light in the food magazine being re-illuminated, after which time a new trial can be initiated by a nose-poke in the food magazine. A behavioural session finished when either a total of 100 trials (not including premature trials) was completed, or 30 minutes had elapsed.

This version of the 5-CSRTT is therefore self-paced: rats are not required to carry out any specific behaviour when waiting for the stimulus light. However, it has been observed that during the waiting period rats leave the food magazine before stimulus presentation and engage in “scanning” behaviour, where they turn to look at the stimulus lights in turn, scanning their heads between each aperture [33], [46], [47], suggesting that rats engage in a consistent pattern of behaviour during the waiting period.

Once stable performance on the 5-CSRTT was achieved, but before electrode implantation, all rats were screened for trait impulsivity as described previously [10]. Screening involved 3 blocks of 5 daily behaviour sessions: in each block rats performed 2 sessions with a 5 second delay, then on the 3^rd^ day the delay was increased to 7 seconds (long delay sessions), followed by 2 more 5-second delay days. Each block was separated by 2 days where rats did not perform the 5-CSRTT. Previous studies (e.g. [10], [11], [34]–[37], [48]–[51]) have defined a sub-population of rats as “highly impulsive”, based on the number of premature responses rats make on the long delay sessions, which increase demand on action restraint. The criterion for high impulsivity was 50 or more premature responses on each of the three long delay sessions. Thus, in highly impulsive rats, a large number of premature responses are made, despite negative consequences (i.e. a reduced opportunity to obtain food reward). Of the 17 rats used in this study, 11 met this criterion for being considered highly impulsive (Table S14 in File S1).

### Surgery

Following 5-CSRTT training and screening for impulsivity, animals were anaesthetised with isoflurane (IsoFlo, Abbott) and, using standard stereotaxic techniques, two custom-fabricated silicon probes [52], [53] were implanted bilaterally into the NAcb and PFC (stereotaxic coordinates relative to bregma in mm: NAcb; anterior-posterior, +1.8, medio-lateral +/−0.8, dorso-ventral −7.0; mPFC: anterior-posterior, +3.0, medio-lateral +/−0.6, dorso-ventral −5.0; based on the atlas of Paxinos and Watson [54]. NAcb probes, of length 10.6 mm, had 4 shanks spaced 0.8 mm, 1.6 mm and 0.8 mm apart with 4 recording sites spaced 0.4 mm apart (total of 16 sites per probe); PFC probes had two shanks spaced 1.2 mm apart with 7 recording sites spaced 0.6 mm apart (total of 14 sites per probe, shank length 7.4 mm).

At the end of the experiment, rats were overdosed with sodium pentobarbital (1.5 mL, 200 mg/mL i.p., Dolethal, Vetoquinol UK) and transcardially perfused with 4% paraformaldehyde. Brains were extracted, sectioned on a freezing microtome, stained with cresyl violet, and electrode positions reconstructed relative to the deepest points of the individual probe tracks, which were clearly visible in the prepared histology (Figure 1A and B).

### Neurophysiological recordings

LFPs were recorded using a wireless telemetry unit ([55], [56] Triangle Biosystems, NC, USA), with further amplification (×12000) and filtering performed (band-pass 1–500 Hz; AM4000; AM systems, WA, USA) prior to analogue-to-digital conversion at a rate of 1.5k Hz or 25 kHz. All recordings were referenced to a stainless steel skull screw (impedance ∼400 Ohms) implanted over the midline cerebellum. Two coloured LEDs on the recording headstage allowed tracking of the animal's location and movement (VideoBench, Datawave Technologies, CO, USA). Video tracking data was sampled at 25 Hz. After recovery from surgery LFPs were recorded daily during 5-CSRTT performance in a total of 83 sessions (7907 trials, see Tables S3 and S4 in File S1 for complete number of sessions and trials recorded in all rats). Recording sessions finished when either 100 trials (excluding premature trials) had been completed, or 30 minutes had elapsed.

### Behavioural analysis

In each trial we defined the time the rat began waiting (typically engaging in “scanning” behaviour) using video-tracking of the LEDs on the recording headstage. This event (“wait-start”) was defined as the first time following the start of a trial where the rat's head left a rectangular area surrounding the food magazine (Figure 1C). This event was therefore necessarily present in all trials which ended in a correct, incorrect or premature response (i.e. all trials analysed). The latency to wait-start on each trial was calculated as (time of wait-start) – (time of trial start), (Figure 1D).

### Data analysis

Data were analysed using custom-written MATLAB (The Mathworks, MA, USA) and R [57] scripts. LFPs were downsampled to 250 Hz before further analysis, using the MATLAB function *decimate*. We identified the presence of an electrical artefact generated by state changes in operant box lighting, time-locked to nose-poking and food magazine entry. In order to mitigate this artefact, prior to analysis, LFP signals were pre-processed with a wavelet based artefact removal algorithm [58] which removed large amplitude artefact components from the LFPs.

Power spectral density (PSD) estimates were calculated over the whole 30 minute recording session using the multi-taper method implemented in the Chronux toolbox (www.chronux.org, [59]), using the function *mtspecgram*c with tapers [2.5 4], a frequency range of 1 to 90 Hz and a time window of 5 seconds, advanced in steps of 5 seconds, averaging in the time domain to give the PSD. The PSD was then converted to decibels by taking 10 * log10 (PSD). Re-referenced PSD estimates were calculated by subtracting from each raw signal the mean of the raw signals recorded simultaneously from all electrodes placed in the same region in that rat. In order to produce PSD estimates on comparable scales before and after re-referencing, given re-referencing reduced signal amplitudes, before time-frequency analysis the raw or re-referenced LFP signals were converted to z-scores by subtracting their mean and dividing by their standard deviation.

**Figure 2:**
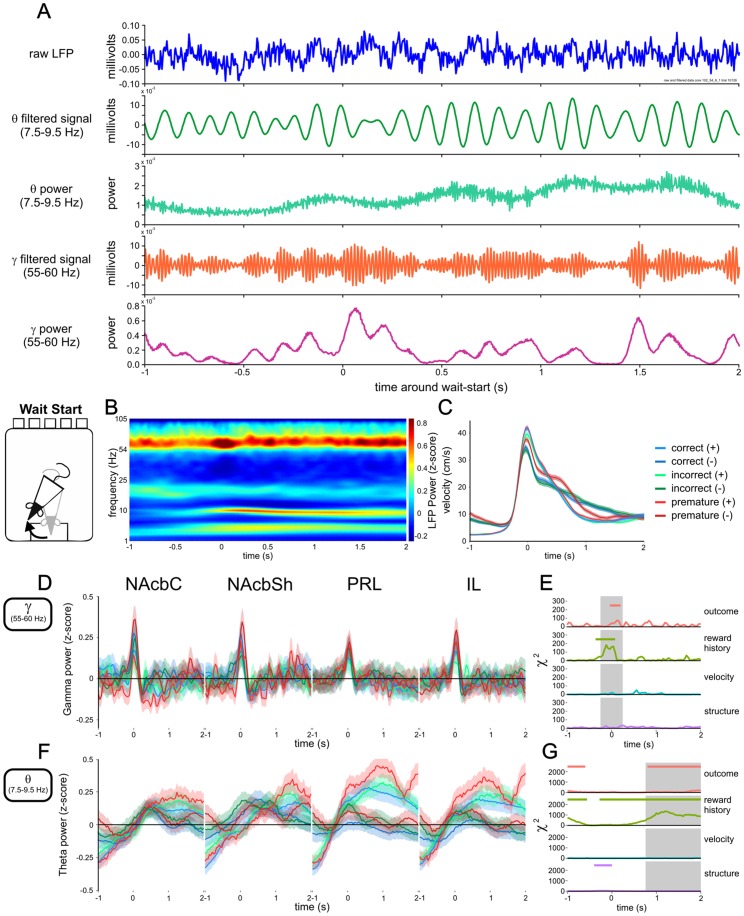
Gamma60 and theta LFP power at the start of waiting. A) Representative example of raw LFP data, filtered gamma60 and theta components of the signal, and the power of gamma60 and theta in the signal. B) Illustrative spectrogram of z-scored LFP power from 1 second before, to 2 seconds after the start of waiting behaviour, between 1 and 105 Hz (logarithmically spaced), for all correct, previously rewarded trials recorded in the NAcbC. C) Average peri-wait-start velocity traces, split by upcoming trial outcome and previous reward. Solid lines represent the mean of all trials (see Table S4 in File S1 for numbers of trials), shaded areas represent the standard error of the mean (SEM). D) Z-scored gamma60 LFP power around wait-start for trials ending in correct, incorrect, or premature responses, split by outcome of previous trial (in legend (+): previously rewarded trials, (−): previously non-rewarded trials). E) Model statistics for the effect of upcoming trial outcome, previous reward, velocity, and brain region on instantaneous gamma60 power. Regions with statistically significant effects (*P*<0.01, bonferroni corrected for multiple comparisons) are highlighted with coloured horizontal bars. Regions used for windowed analysis are highlighted in grey. F) Z-scored theta LFP power around wait-start. Solid lines represent the mean of all trials. Shaded areas represent the SEM. G) Model statistics for the effect of upcoming trial outcome, previous reward, velocity, and brain structure on instantaneous theta power.

Example signals (Figure 2A) were filtered using two-way least-squares FIR filters (implemented as the function *eegfilt* in the EEGLAB toolbox http://sccn.ucsd.edu/eeglab/ [60]).

Time-frequency analysis was performed using the continuous wavelet transform (CWT), convolving LFP signals with complex morlet wavelets using the MATLAB function *cwt* (wavelet ‘cmor1-3’, [bandwidth parameter 1, centre frequency 3 Hz], details of the MATLAB implementation of the continuous wavelet transform can be found at http://www.mathworks.co.uk/help/wavelet/gs/continuous-wavelet-transform.html).

**Figure 3:**
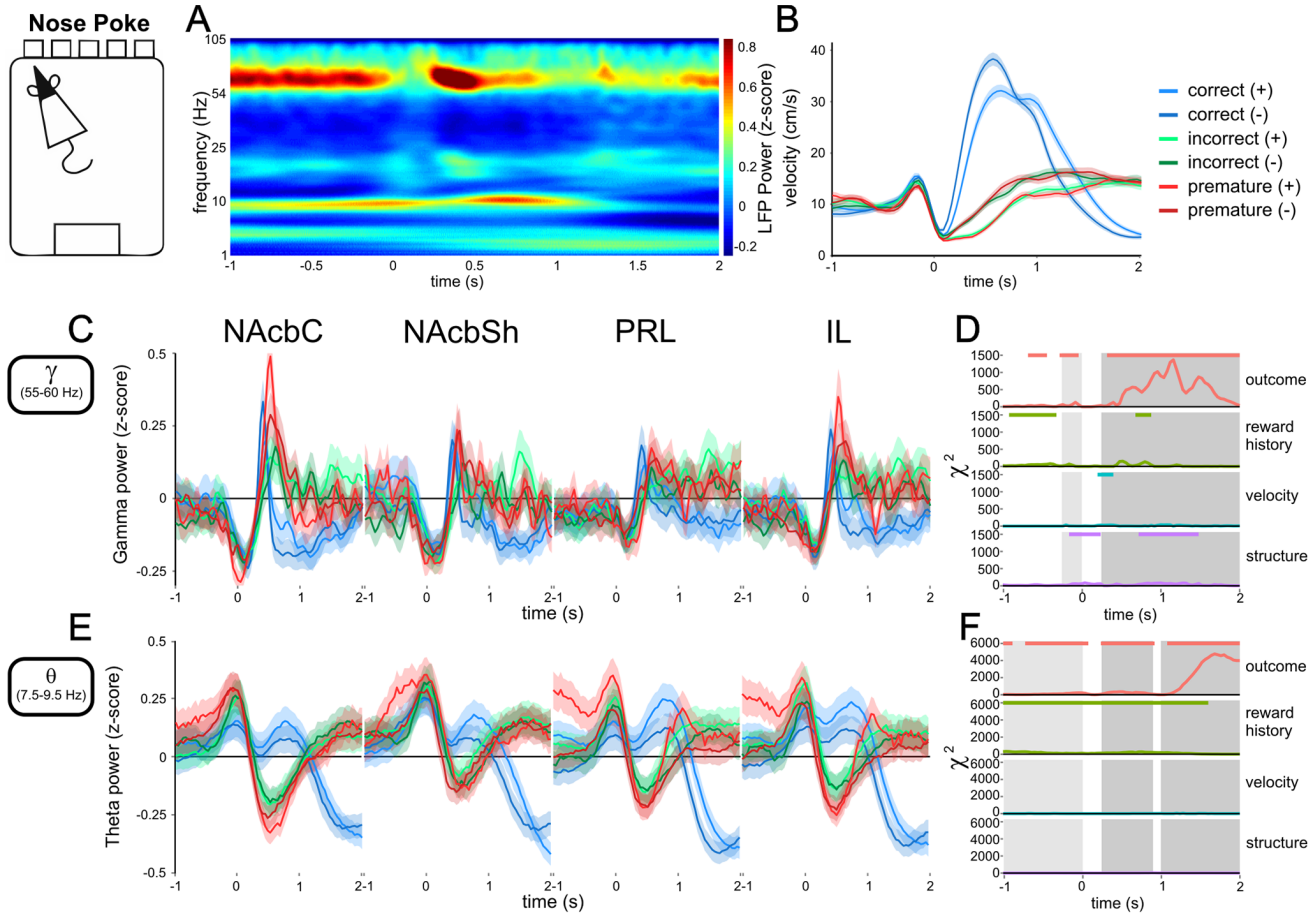
Gamma60 and theta LFP power correlates with trial outcome following responding. A) Spectrogram of z-scored LFP power from 1 second before, to 2 seconds after nose-poking, between 1 and 105 Hz, for all correct, previously rewarded trials recorded from the NAcbC. B) Average peri-nose-poke velocity traces, split by upcoming trial outcome and previous reward. C) Z-scored gamma60 LFP power around nose-poking. Solid lines represent the mean of all trials. Shaded areas represent the SEM. D) Model statistics for the effect of upcoming trial outcome, previous reward, velocity, and brain region on instantaneous gamma60 power. E) Z-scored theta LFP power around nose-poking. Solid lines represent the mean of all trials. Shaded areas represent the SEM. F) Model statistics for the effect of upcoming trial outcome, previous reward, velocity, and brain region on instantaneous theta power. Windows before nose-poking are highlighted in light grey. Windows following nose-poking are highlighted in darker grey.

Illustrative spectrograms (Figure 2B and Figure 3A) were produced as follows: for each 30 minute recording session a spectrogram was calculated using the CWT at each of a series of logarithmically spaced frequencies between 1 and 110 Hz. The mean and the standard deviation (SD) of the spectrogram over time was calculated (giving the average power spectral density, and its SD), and 1/f curves were fit to both (curves of the form y = a*x∧b were fit using the *fit* function in the MATLAB curve fitting toolbox). The curve fit to the mean PSD was subtracted from windows of the spectrogram around the wait-start or nose-poke events in each behavioural trial, and the windowed spectrogram was then divided by the curve fit to the signal SD. This spectrogram was designed to illustrate changes in spectral power over time during 5-CSRTT behaviour, de-emphasising low frequency components, and therefore allowing the whole spectrogram to be illustrated on a common colour axis. However, this data was not used for further quantitative analysis.

For quantitative time-frequency analysis of LFP power in the gamma and theta frequency bands (Figure 2D–G and Figure 3C–F), peri-event LFP power in these bands (theta: 7.5–9.5 Hz, gamma60: 55–60 Hz, selected as the frequencies of peak power from the PSD) was calculated using the CWT, and z-scored by subtracting the mean and dividing by the SD of power in that frequency band calculated over the whole 30 minute recording session.

### Phase-amplitude coupling

Various methods have been proposed to assess phase-amplitude coupling (PAC) [61]–[66], and there is little consensus to which method is optimal. Onslow *et al.*, (2011) suggest that most methods produce similar results, but that the envelope-to-signal correlation (ESC) measure proposed by Bruns & Eckhorn (2004) is more accurate over short time windows of data, which best matched our experimental objectives. The ESC method measures PAC as the Pearson's correlation between the amplitude envelope of the filtered high-frequency signal and the filtered low frequency signal (the signal providing phase information): ESC*_(pf, af)_*  =  corr(FiltL, AmpH), where *pf* is the phase-giving frequency, *af* is the amplitude giving frequency, FiltL is the filtered low frequency signal and AmpH is the amplitude of the filtered high frequency signal.

**Figure 4:**
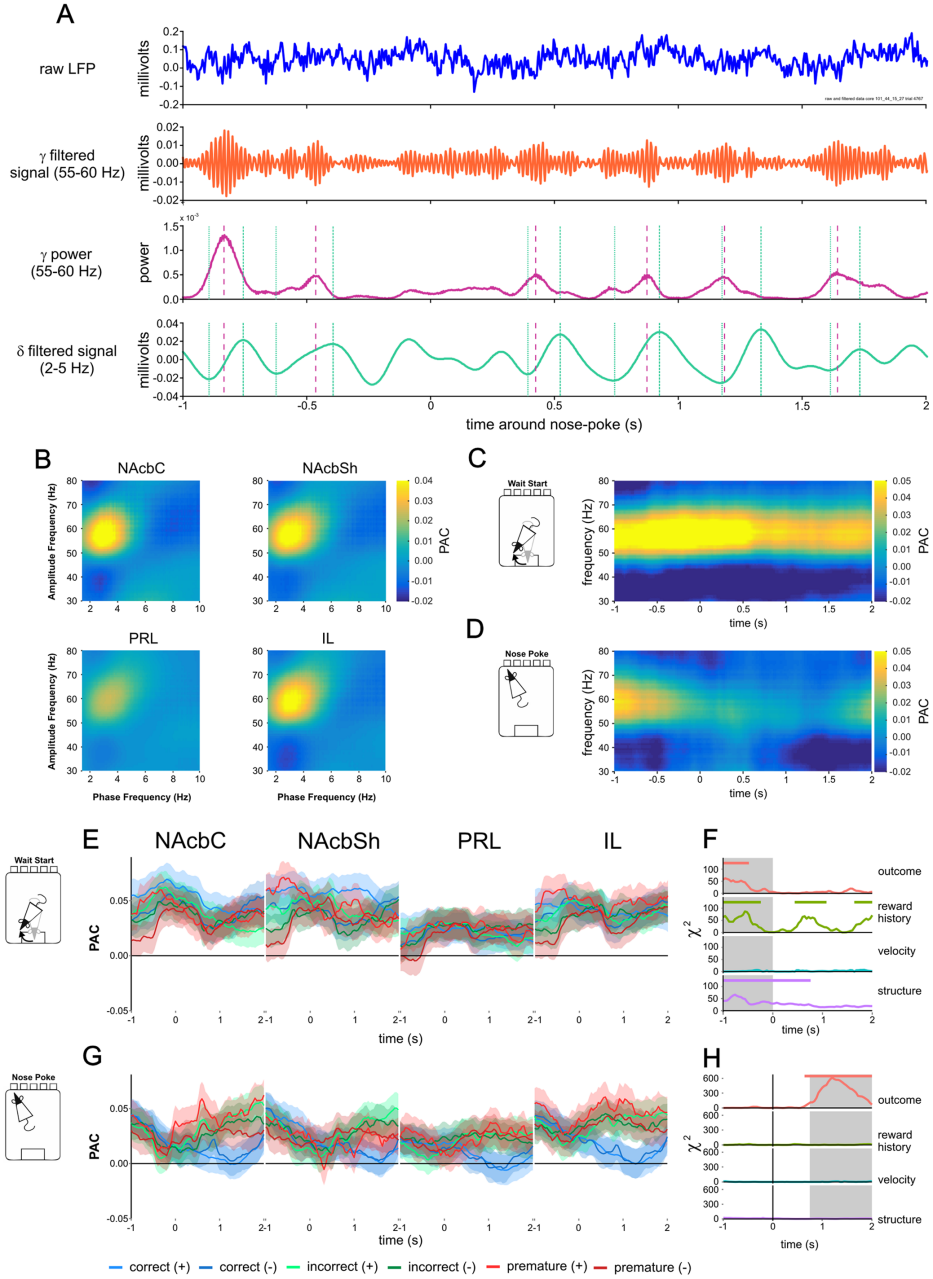
Corticostriatal gamma60-delta PAC during 5-CSRTT performance. A) Example of raw and filtered LFP data showing PAC. Vertical dashed purple lines indicate local gamma60 power maxima; vertical green lines indicate local delta peaks and troughs. B) Phase-amplitude coupling between low (phase giving) and high frequency (amplitude giving) oscillations in PFC and NAcb calculated over whole 30 minute recordings. PAC peaked between gamma60 oscillations and a 2–3 Hz delta oscillation, and was weaker in PRL than other regions. Note that PAC was calculated with amplitude and phase data taken from the same electrode. C) PAC between 30–80 Hz high frequency oscillations and a 2.75 Hz delta oscillation around wait-start for all correct, previously rewarded trials recorded from NAcbC electrodes. D) PAC between 30–80 Hz and 2.75 Hz delta oscillations around nose-poking for all correct, previously rewarded trials recorded from the NAcbC. E) Gamma60-delta PAC around wait-start. Solid lines represent the mean of all trials. Shaded areas represent the SEM. F) Model statistics for the effect of upcoming trial outcome, previous reward, velocity and brain region on instantaneous gamma60-delta PAC around the wait-start alignment event. G) Gamma60-delta PAC around nose-poking. Solid lines represent the mean of all trials. Shaded areas represent the SEM. H) Model statistics for the effect of upcoming trial outcome, previous reward, velocity, and brain region on instantaneous gamma60-delta PAC around nose-poking.

PAC was calculated using MATLAB code from the toolbox of Onslow *et al.*, (2011) (http://www.cs.bris.ac.uk/Research/MachineLearning/pac/). The presence of PAC was assessed by calculating the ESC measure over the whole 30 minute task recording, using phase-providing low frequencies between 1.5 and 10 Hz and amplitude-providing high frequencies between 30 and 80 Hz (Figure 4B). Phase and amplitude data were taken from recordings from the same electrode. It has been suggested that using amplitude and phase data from the same electrode can produce artefactual coupling [68]. To exclude this possibility PAC was recalculated for each recording session taking amplitude and phase from difference electrodes located in the same brain region. PAC was calculated between all possible pairs of phase and amplitude-giving electrodes in each session, and then averaged, to give one value per structure per recording session. This analysis produced the same pattern of PAC as calculating PAC using amplitude and phase data from the same electrode (Figure S5A), so the single electrode method was used for all further PAC analyses to give a dataset of the same size as the LFP analysis.

Changes in PAC during task performance were analysed using a moving window approach: PAC between a low frequency of 2.75 Hz (selected as the peak low frequency from the whole session plots) and high frequencies between 30 and 80 Hz for colour plots in Figure 4C and D, or 55–60 Hz for linear plots (Figure 4E and G), was calculated over a 1 second window, advanced in steps of 0.02 seconds.

### Statistical analysis

Multiple, potentially interacting factors, including brain region, trial outcome, previous reward, and velocity could influence LFP measures during 5-CSRTT behaviour, and our dataset had a hierarchical and unbalanced structure (electrodes nested in rats, with rats contributing different and unequal numbers of trials and recording electrodes). Simply grouping all available trials across all electrodes and rats without accounting for this structure violates the assumption of independence between observations required for standard statistical tests, and increases the type 1 error rate through pseudoreplication [69], [70]. Therefore, we used linear mixed models (LMMs, also known as multi-level, or hierarchical, models) to analyse behavioural and LFP data, using random effects structures accounting for this hierarchical data structure.

For behavioural data the non-independence of values derived from the same rat was controlled by including the identity of the rat contributing each data point as a random factor in the LMM. For peri-event LFP analysis, the factors correlating with changes in LFP power or PAC were investigated by fitting a LMM to the LFP measure at every time point in a window from 1 second before to 2 seconds after the event (this window was selected to focus on LFP changes linked to the behavioural event in question, rather than including signal which could be modulated by preceding or subsequent events), with movement velocity, trial outcome, previous trial reward and brain region as independent variables. As velocity data was sampled at 25 Hz, LFP data was also downsampled to 25 Hz for analysis (using linear interpolation, MATLAB function *interp1*). LMMs were fit with as maximal a random effects structure as possible [71] (in standard Wilkinson notation (velocity|ratID/channel ID). To compensate for multiple comparisons over time, we adopted a conservative threshold for significance (*P*<0.01, Bonferroni corrected, so for 3 seconds of peri-event data sampled at 25 Hz, the threshold for significance was adjusted to 0.00013), with the additional stipulation that to be considered meaningful an epoch of significance for an effect must have a duration exceeding 0.2 seconds.

Windows of interest were identified as epochs with significant main effects related to 5-CSRTT events, and the average LFP power in each window was further analysed with a LMM including all interactions terms. To correct for analysing multiple time windows *P-*values were Bonferroni corrected by multiplying by the number of analysis windows (9). LMMs were fit using the *lmer* function from the *lme4* package in R [72]. *P*-values were calculated using orthogonal contrasts and type-3 sums of squares using Wald χ^2^ tests, and model contrast *t* values were calculated using Satterthwaite's approximation for degrees of freedom, using the *lmerTest* R package. Post-hoc tests were performed with the *glht* function in the *multcomp* R package, using Tukey all-pair comparisons and Bonferroni corrections for multiple comparisons.

We tested whether LFP data from the window around the wait-start event could be used to predict upcoming premature responses using a generalised linear mixed model (GLMM) with a binomial distribution and logit link function (*glmer* and *predict* methods from *lme4*, equivalent to a logistic regression model with random effects terms). A binary dependent variable (premature or non-premature [either correct or incorrect]) was used. To give a dataset with the same number of data points as there were physical trials, we took trials from rats with electrodes in both NAcbC and PRL (n = 15, a total of 6672 trials), and for each trial took the average gamma60 power across all NAcbC electrodes in the window [−0.25 0.25] around wait start, the average theta power across all PRL electrodes in the window [0.75 2] around wait-start, and the average PAC across all NAcbC electrodes in the window [−1 0] before wait-start, based on those LFP analysis windows with significant effects of outcome (Figure 2, 3 and 4 and Figures S2, S3 and S5). We focused on LFP data aligned to the wait-start event as a predictive model would be most informative if it allowed premature responses to be predicted as early as possible within a trial: while we also found significant effects of upcoming trial outcome on gamma60 and theta power immediately before nose-poking (Figure 3 and Figure S3), this time window would include times where the rat had already began making the nose-poke movement, so could reflect preparatory or movement-related processes.

GLMMs fits to the data were compared using likelihood ratio tests (that is, testing if the log-likelihood of two models are significantly different, using the *anova.merMod* method from *lme4* [e.g. *anova(model1, model2, test  =  “LRT”]* [73]).

To determine how accurately models could predict previously unseen trial data, we used leave-one-out cross validation (LOOCV), repeatedly refitting the model to the full dataset except one trial, and then using the model to predict the probability of the left-out trial being a premature response. We chose the GLMM approach, rather than other classification techniques as other approaches assume that each element in the training and test datasets are independent and identically distributed. However as out dataset included multiple trials from the same animals, this assumption was violated, which could be addressed using the GLMM approach by including the identity of the rat contributing each trial as a random factor.

Model classification performance after LOOCV was measured using the receiver-operator characteristic (ROC) approach. This method is advantageous in assessing classification performance in situations with unbalanced numbers of data in each group (premature responses were much less frequent than non-premature responses) as it assesses the performance of a model over a range of threshold values (i.e. threshold values of predicted probability at which to classify responses as premature). The area under the ROC curve (AUC) is a measure of classifier performance based on the area under the curve produced by plotting the classification true positive rate against the false positive rate (also described as hit and miss rates i.e. true positives are true premature trials correctly classified as prematures, and false positives are true non-premature trials incorrectly classified as prematures) at different threshold values. An AUC of 0.5 indicates an uninformative classifier (the true positive and false positive rates are equal). The AUC can also be considered as equivalent to the probability that, if given a randomly selected true premature trial and a true non-premature trial, the model would give the true premature sample a higher probability of being a premature than the true non-premature sample. In addition to the ROC curve, we also calculated the model accuracy as the (number of true positives) + (number of true negatives)/(number of true positives) + (number of false positives) + (number of true negatives) + (number of false negatives) over all possible classification threshold values. AUC and classification performance measures were calculated using the *ROCR* R package [74]).

In addition to the likelihood ratio tests used to assess whether adding LFP data improved model fit, differences in classification performance after LOOCV was assessed directly using a bootstrapping test, implemented in the *pROC* R package [75]. In this test 2000 samples were drawn from the LOOCV prediction data for each model. For each sample, the AUC for each model, and the difference in AUC between models, was calculated. A test statistic was then calculated as D =  (AUC_model1_ – AUC_model2_)/(standard deviation of bootstrap AUC values). D was then compared to the normal distribution (two –tailed) to give a *P* value.

To test whether rat's impulsivity screening scores related to 5-CSRTT LFP measures, we extracted variables (Table S15 in File S1) from the LFP windows of interest that we identified around the wait-start and nose-poke events. Ten LFP variables derived from gamma60 and theta power and gamma60-delta PAC were extracted in 15 rats with electrodes in both PRL and NAcbC. Ten variables were selected because we had 11 highly-impulsive rats and Principal Component Analysis requires fewer variables than data points. Variables were taken from the NAcbC and PRL for balance, and were selected based on the peri wait-start and nose-poke analysis window's largest effects of outcome or previous reward. For each variable, the LFP measure in the window of interest was averaged over all trials in each rat, giving one value per variable per rat. Principal Component Analysis (PCA) was used to extract features from the set of variables (PCA was performed using the *principal* function in the *psych* R package). A PCA without factor rotation indicated 4 factors explained >95% of the variance in the LFP variance. To improve the interpretability of factor loading on the LFP variables, the PCA was then repeated using oblique rotation (oblimin) targeting 4 factors.

## Results

### Behavioural data

We investigated corticostriatal LFPs during waiting behaviour by implanting microelectrodes in the medial PFC (prelimbic and infralimbic cortices [PRL and IL respectively]) and the NAcbC and NAcbSh (Figure 1A and B, Table S1 in File S1) of rats trained to perform the 5-CSRTT. To investigate 5-CSRTT related LFP activity, we focused on 2 key task events: (i) the time the rat left the food magazine and began scanning and waiting behaviour; defined as “wait-start”; and (ii) the time the rat made a nose-poke response in one of the 5 target apertures.

Lesions of NAcb have previously been shown to affect 5-CSRTT performance exclusively during trials when rats have made errors on the preceding trial [76]. We therefore investigated whether previous trial outcome influenced behaviour on the subsequent trial. Correct responses made up a significantly smaller proportion of all trials where the rat was previously non-rewarded (or equivalently, rats were more likely to make a correct response when the previous trial was also correct) (*χ* ^2^_1_ = 29.980, *P*<0.001, linear mixed model). In contrast to correct responses, premature responses were significantly increased as a proportion of trials when the rat was previously not rewarded (*χ* ^2^_1_ = 32.82, *P*<0.001). Therefore previous trial outcome influenced the behavioural endpoint of the subsequent trial.

It has also been suggested that making errors affects reaction times on subsequent trials [77]. We investigated whether current trial outcome or previous reward influenced the latency of rats to move from the start of a trial to wait-start (Figure 1D, see Table S2 in File S1 for average latency data, and data for each rat, and Table S3 in File S1 for total numbers of trials analysed). We found significant effects of outcome and previous reward on wait-start latency (*χ* ^2^_2_ = 49.860, *P*<0.001 and *χ* ^2^_1_ = 36.565, *P*<0.001, respectively, with no interaction: *χ* ^2^_2_ = 2.134, p = 0.344 [linear mixed model]).

Compared to trials ending in correct responses, trials which ended in incorrect responses were associated with significantly slower latencies to wait-start (t_21.534_ = 2.342, *P* = 0.029), while trials that ended in premature responses were associated with significantly faster latencies (t_12.688_ =  −5.668, *P*<0.001), and trials which were previously non-rewarded were associated with faster movement to wait-start, regardless of the outcome of the upcoming trial (t_16.063_ =  −5.839, *P*<0.001). This effect was true for each upcoming trial outcome: previously non-rewarded trials were associated with significantly faster latencies to move to wait-start on correct trials (z =  −5.839, *P*<0.001), incorrect trials (z =  −4.761, *P*<0.001) and premature trials (z =  −7.865, *P*<0.001).

### Wait-start gamma60 and theta LFP power

We found consistent LFP oscillations in a number of discrete frequency bands (Figure 2A and Figure S1A) during the 5-CSRTT sessions, with gamma60 (55–60 Hz) and theta (7.5–9.5 Hz) being most prominent. Power in these bands was similar following re-referencing of signals to the average of the signal from all electrodes simultaneously recorded in the same brain region (Figure S1B), suggesting these oscillations were local in origin and not exclusively the result of volume conduction from distant oscillators [78]. See Figure 2A for examples of raw and filtered data in these bands.

LFP Power in both the gamma60 and theta frequency bands was influenced by the wait-start event (Figure 2B, D and F, see Table S4 in File S1 for total numbers of electrode-trials analysed). Gamma60 power increased transiently at wait-start (Figure 2D), most prominently in the NAcbC. In contrast, theta power showed a slower increase as the rats began waiting (Figure 2F), which was greater in the PFC compared with the NAcb.

To investigate the factors influencing LFP power around the wait-start event we quantified whether brain region, movement velocity and upcoming or previous trial outcome influenced gamma60 or theta power (Figure 2E and G). Gamma60 and theta frequency LFPs were significantly influenced by previous trial reward, upcoming trial outcome and brain region, but not velocity. In keeping with the latency differences observed between previously rewarded and non-rewarded trials, peak velocity was higher on previously rewarded trials (Figure 2C): here rats were slower to leave the food magazine and thus moved faster to avoid missing the target stimulus.

Focussing on gamma60 power in a window from 0.25 seconds prior to, to 0.25 seconds after the wait-start event we used a linear mixed model to investigate the effects of upcoming trial outcome, previous reward and brain region (and interactions), on changes in gamma60 power (Figure S2A and Table S5 in File S1). We found a significant interaction of upcoming trial outcome, previous reward and brain region (*χ* ^2^_6_ = 25.116, *P* = 0.003), suggesting that the wait-start associated increase in gamma60 power differed between brain structures and upcoming trial outcomes, as well as related to previous trial outcome. The reward history effect was larger in NAcb than PFC (Figure S2A), with trials following errors being associated with greater gamma60 increases at wait-start. We applied the same analysis to changes in theta power in the window from 0.75 to 2 seconds post-wait-start (Figure S2B and Table S6 in File S1). In contrast to gamma60 in the NAcb, in the theta band, the increase in theta power following wait-start was smaller in PFC on trials following errors (previous reward X brain region interaction *χ* ^2^_3_ = 305.929, *P*<0.001). Similar to the gamma60 band, wait-start theta power changes also related to upcoming trial outcome (effect of outcome *χ* ^2^_3_ = 141.975, *P*<0.001 and interaction of outcome and previous reward *χ* ^2^_2_ = 37.418, *P*<0.001). Therefore, LFP power changes in the gamma60 and theta bands around the time rats start to engage in waiting behaviour are differentiated between brain region and past experience, and contain information about upcoming behaviour.

### Nose-poke response gamma60 and theta LFP power

The waiting period in the 5-CSRTT is terminated by a nose-poke response in one of the 5 target apertures. Both gamma60 and theta LFPs in the PFC and NAcb were significantly affected by the outcome of nose-poking (Figure 3A, C–F). In gamma60, particularly in NAcbC, correct responses were associated with a transient decrease, increase and then decrease in power, with the increase peaking around 0.4 seconds following the nose-poke. This transient response was also present in the NAcbSh and IL, but was smaller in PRL. By contrast, following error responses (i.e. incorrect and premature responses), gamma60 power increased, remaining elevated for approximately 2 seconds post nose-poking compared to correct responses (Figure 3C and D).

In the theta band correct nose-pokes were associated with a small increase in power, followed by a larger decrease, whereas error responses were associated with a transient decrease in theta power, followed by increased power. This produced an early period between 0.25 and 0.9 seconds following nose-poking where correct responses were associated with significantly higher theta power, followed by a late period from 1 second post-poke associated with significantly higher theta power following errors (Figure 3E and F). Unlike gamma60, theta power was also significantly influenced by previous reward throughout much of the peri-nose-poking epoch (Figure 3F).

Analysis of average gamma60 power from 0.25 to 2 seconds following nose-poking indicated that outcome explained the most variance in power *χ* ^2^_2_ = 3808.919, *P*<0.001 (Figure S3B, Table S8 in File S1). Gamma60 was also influenced by outcomes before they occurred (effect of outcome on gamma60 power from 0.25 seconds before to nose-poking *χ* ^2^_2_ = 118.646, *P*<0.001, Figure S3A, Table S7 in File S1). Specifically, gamma60 power was reduced prior to premature responses (Figure 3C, Figure S3A). Trial outcome also explained the most variance in the theta band both at a short latency (0.25–0.9 seconds post poke, effect of outcome *χ* ^2^_2_ = 2180.355, *P*<0.001, Figure S3D, Table S10 in File S1), and a longer latency (1–2 seconds, effect of outcome *χ* ^2^_2_ = 5003.159, *P*<0.001, Figure S3E, Table S11 in File S1) post nose-poke. In the 1 second preceding nose-poking theta power was also correlated with upcoming behaviour (Figure S3, Table S9 in File S1), with theta power being elevated preceding premature responses, particularly in PFC, as was observed relative to the start of waiting (Figure 2F and G and Figure S2B). Peri-nose-poke gamma60 and theta power was also significantly affected by brain region, previous reward and impulsivity phenotype (Figure S3, Table S7–11 in File S1).

We found only one brief epoch in gamma60 power where there was a main effect of velocity on LFP power (Figure 3D), suggesting that predominantly our results were not directly related to movement. Rats moved faster following correct nose-pokes (Figure 3B), with the average latency for rats to move from nose-poking to the food magazine (where in the case of correct responses they received a food reward) being 1.34±0.22 seconds (mean ± SEM) for correct responses, compared to latencies of 5.02±1.14 seconds following incorrect responses, and 5.17±1.41 seconds following premature responses. There was a significant effect of trial outcome on latency (*χ* ^2^_2_ = 227.957, *P*<0.001), with no effect of previous reward (*χ* ^2^_1_ = 0.094, *P* = 0.760) or interaction between current trial outcome and previous reward (*χ* ^2^_2_ = 2.785, *P* = 0.248. Therefore, outcome-related LFP changes in the gamma60 and theta bands occurred at latencies preceding either reward receipt or the rat returning to the food magazine at the end of a punishment timeout.

However, given the differences in behaviour following correct and error nose-pokes, the LFP changes we observed in this epoch might have related to different behaviours the rats engage in subsequent to nose-poking. To address this we plotted z-scored gamma60 and theta power around nose-poking, with trials binned by the latency between nose-poking and returning to the food magazine on that trial (Figure S4). The large differences in latencies between correct and error trials meant that not all bins at short latencies following error trials contained trials, and longer latency bins following correct trials contained only a small number of trials, and therefore had increased variance. However, we found that the increase in gamma60 power following nose-poking did not vary based on magazine latency, whereas following correct responses the subsequent dip in gamma60 power appeared to occur relative to the time of arrival at the food magazine. In the theta band the late decrease in theta power following correct nose-pokes occurred after rats returned to the food magazine, whereas following error responses, theta power decreased immediately, regardless of magazine latency. Therefore, while we did not observe that changes in LFP power were associated with movement per se, some outcome-related differences in LFP power could relate to the different behaviours rats engaged in subsequent to nose-poking, particularly those occurring at least 1 second after nose-poking.

### Gamma-Delta phase-amplitude coupling

Based on previous reports of phase-amplitude coupling (PAC) between low-frequency oscillations and gamma oscillations in the striatum and PFC [67], [79]–[83], [68], we investigated whether LFPs recorded in PFC and NAcb showed PAC. Analysing whole 30 minute recording session data we found PAC between a low-frequency 2–5 Hz delta oscillation and the 55–60 Hz gamma60 oscillation in NAcb and PFC (Figure 4B, see Figure 4A for plots of raw and filtered data). This gamma-delta PAC differed between brain region (effect of region *χ* ^2^_3_ = 32.709, *P*<0.001), with PAC being lower in PRL compared to NAcbC (z =  −4.653, *P*<0.001), NAcbSh (z =  −4.991, *P*<0.001) and IL (z =  −3.192, *P* = 0.008). PAC was also re-calculated by taking phase and amplitude data from different electrodes placed in the same brain region, across all possible pairs of electrodes. This analysis produced similar patterns of PAC (Figure S5A), suggesting PAC was widespread throughout the NAcb and PRL.

During 5-CSRTT performance gamma-delta PAC was influenced by both waiting and nose-poke responses (Figure 4C–H). PAC reduced throughout waiting in the NAcbC (Figure 4C and D), with other structures showing different patterns (Figure 4E, Figure S5B and C, Table S12 and S13 in File S1). Notably, PAC was significantly influenced by previous reward before and during waiting (Figure 4E). Following nose-poking, similar to gamma60 power, the largest influence on PAC was trial outcome: following correct nose-poke responses, PAC decreased compared with error responses. In the window from 0.75 to 2 seconds post nose-poke there was a significant effect of trial outcome on PAC *χ* ^2^_2_ = 610.629, *P*<0.001, an effect which was modified by brain region and previous trial outcome (Figure 4G and H, Figure S5B, Table S13 in File S1). Similar to LFP power in the gamma and theta bands, PAC was not significantly influenced by velocity.

### Predicting impulsive responses from LFP data

**Figure 5:**
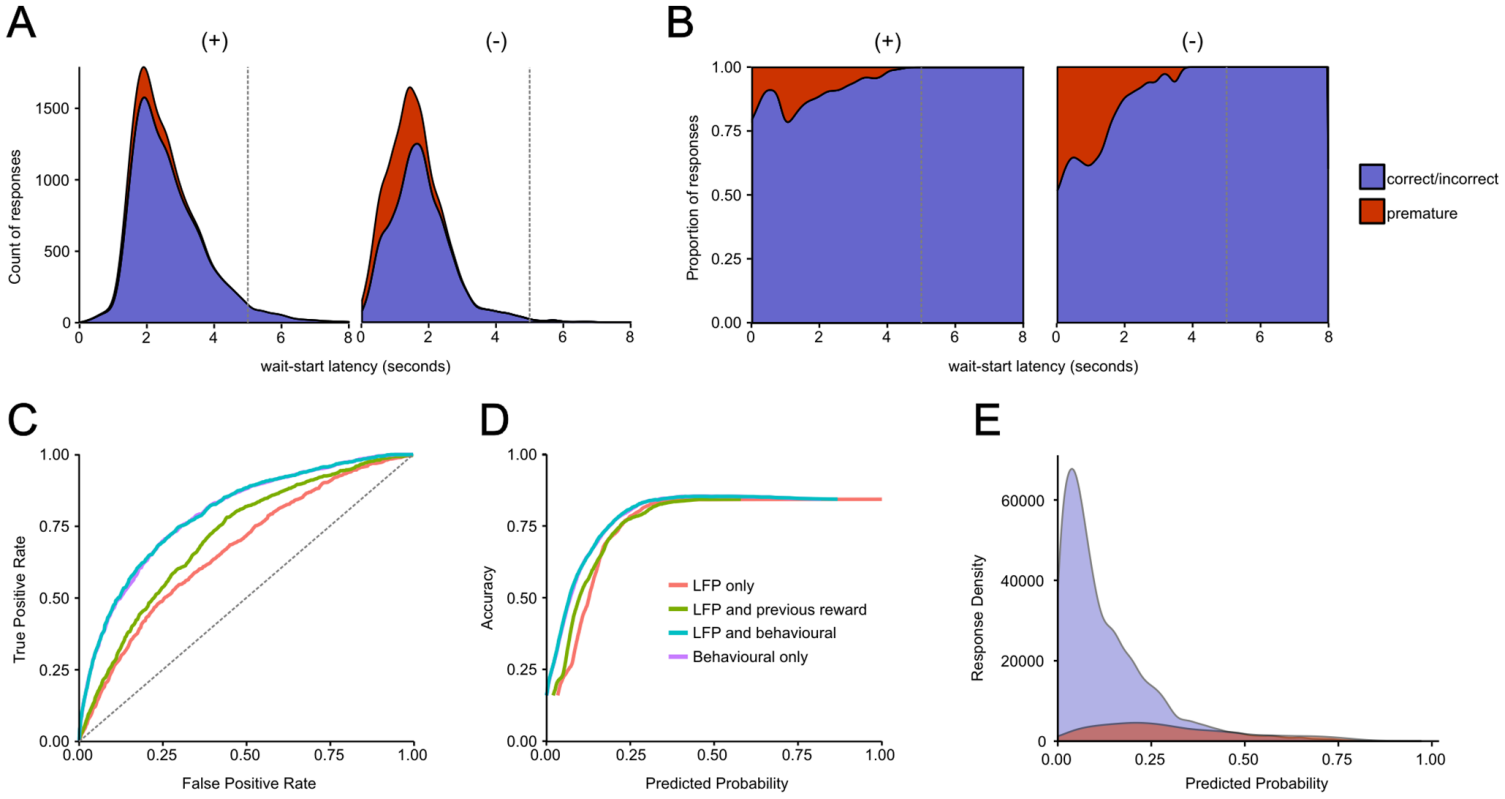
Predicting upcoming impulsive responses. A) Stacked distribution of premature and non-premature (i.e. correct and incorrect) responses as a function of latency of rats to move to wait-start, divided into trials where the previous trials was rewarded (+), or non-rewarded (−). Time zero is the start of the trial, a vertical grey line represents the time of stimulus light presentation (or in the case of premature responses, the time the stimulus light would have been presented). B) Distribution of premature and non-premature responses as depicted in A, represented as a proportion of all responses. C) Receiver-operator characteristic curve for models predicting upcoming premature responses based on leave-one-out cross-validation results. The diagonal grey line represents an uninformative classifier. D) Plot of model accuracy ([number of true positives] + [number of true negatives]/[number of true positives] + [number of false positives] + [number of true negatives] + [number of false negatives]) against threshold predicted probability value. E) Distribution of predicted probabilities for true premature and non-premature trials from the full (behaviour plus LFP) model. The area under each curve is equal to the total number of trials.

Given our finding that LFP power and PAC around the wait-start behavioural event was significantly influenced by upcoming trial outcome, we asked whether it was possible to use LFP and behavioural data to make a model which predicted upcoming premature responses (compared to correct and incorrect responses – trials where the rat waited for the stimulus light successfully). To give a dataset with the same number of data points as there were physical trials, we analysed trials from rats with electrodes in both NAcbC and PRL (n = 15), giving 6672 trials, of which 1048 (15.71%) ended in premature responses. As was the case with the full dataset, premature responses were more likely when rats made an error on the previous trial (732 premature responses were made when the rat previously made an error, compared to 316 premature responses made following correct responses; by contrast 3238 correct and incorrect responses were made following correct trials, and 2386 were made following error trials [Pearson's Chi-squared test χ^2^_1_ = 265.750, *P*<0.001]). Similarly, as with the full dataset, premature responses and trials were the rat previously made an error were associated with shorter latencies to wait-start (effect of outcome χ^2^_1_ = 40.360, *P*<0.001, effect of previous reward χ^2^_1_ = 45.980, *P*<0.001, with a non-significant interaction χ^2^_1_ = 0.625, *P* = 0.429, Figure 5A and B).

A model containing the gamma60 power in the NAcbC around wait-start (in the window from 0.25 seconds before to 0.25 seconds after wait-start) and PRL theta power after wait-start (in the window from 0.75 to 2 seconds following wait-start) was significantly better at predicting upcoming premature responses than an intercept-only model including the random effects term, (χ^2^_3_ = 18.508, *P*<0.001, likelihood ratio test of model fit). This LFP-based model gave an AUC after leave-one-out cross-validation of 0.6694 (Figure 5C and D). Adding peri-wait-start PAC data did not improve model fit (χ^2^_4_ = 7.027, *P* = 0.135), so PAC data was not used for further analysis.

Given that the effects of upcoming trial outcome on LFP power interacted with previous trial outcome (Table S5 and S6 in File S1), we tested whether adding previous reward improved model fit. This was indeed the case (χ^2^_4_ = 196.480, *P*<0.001), giving a model AUC of 0.7134 (Figure 5C and D). Trials which ended in premature responses were also associated with shorter latencies for the rat start waiting, and adding this latency improved model fit (χ^2^_8_ = 584.600, *P*<0.001), giving a model AUC of 0.7964 (Figure 5C–E). Given that behavioural data appeared to be a strong predictor of upcoming response type, we asked whether behavioural data alone (previous trial reward and wait-start latency on the current trial) was a predictor of upcoming premature responses. The behaviour – only model performed significantly better than the intercept-only model (χ^2^_3_ = 762.950, *P*<0.001), with an AUC of 0.7947. Given the strong performance of the behaviour-only model we asked whether the LFP data made a significant contribution to improving model fit over the behavioural data alone: it could simply be the case that the LFP data reflects behavioural events and therefore contains no additional information about upcoming behaviour. The full LFP plus behavioural model fit the data significantly better than the behavioural only model (χ^2^_12_ = 36.636, *P<*0.001).

However, when the AUC values produced by the behaviour-only and LFP-behavioural models were directly compared using a bootstrapping test the combined model did not produce a significantly greater AUC value (D = 1.0596, *P* = 0.2893). Therefore, while adding LFP data significantly improved model fit to the trial outcome data, this did not produce a significant improvement in trial outcome classification performance compared to that provided by behavioural data alone.

### Relating trait impulsivity to LFP data

In addition to investigating whether corticostriatal LFPs could be used to predict upcoming impulsive behaviours, we investigated whether these LFPs contained information about individual rat's trait level of impulsivity. It has been previously demonstrated that rats exhibit natural variation in premature responding, and that these behavioural differences are accompanied by neurobiological differences, which may be causal in impulsivity (e.g. [10], [35]–[37], [48], [49]). We therefore investigated whether any of the LFP windows we identified as being modulated during 5-CSRTT performance were influenced by trait impulsivity. Before electrode implantation, rats were screened for impulsivity during 3 sessions where the delay before cue presentation was increased from 5 to 7 seconds (see *Materials and Methods*). The average number of premature responses made during these sessions was then taken as the rat's impulsivity score (Table S14 in File S1).

First we investigated whether LFP power or PAC in the peri-event windows of interest we identified correlated with rat's impulsivity screening score (see Table S15 in File S1 for details of the LFP variables used and Figure S6A). LFP variables were calculated from NAcbC and PRL as these structures contributed the largest numbers of electrodes. In order to have each rat include data from both NAcbC and PRL, 2 rats which only had electrodes in PRL were excluded.

Several LFP variables appeared to have correlations with impulsivity screening scores (e.g. post-poke gamma60 power in the NAcbC), but with a number of outliers. Rats which make more than 50 premature responses in each of the 3 screening sessions have been described as “highly impulsive” (HI), and have been demonstrated to show a discrete set of neurobiological and behavioural differences compared to rats showing low levels of impulsivity in screening (e.g. [10], [11], [34]–[37], [48]–[51]). We therefore investigated whether there was a relationship between LFP variables and impulsivity screening score specifically in rats whose screening scores met the criteria for high impulsivity (n = 11). Two of the “non- highly impulsive” rats did not have electrodes in both PRL and NAcbC, leaving only 4 rats which were not “highly impulsive”. When the highly impulsive group of rats was analysed alone, LFP variables were found which significantly correlated with impulsivity screening scores (Figure S6A).

**Figure 6:**
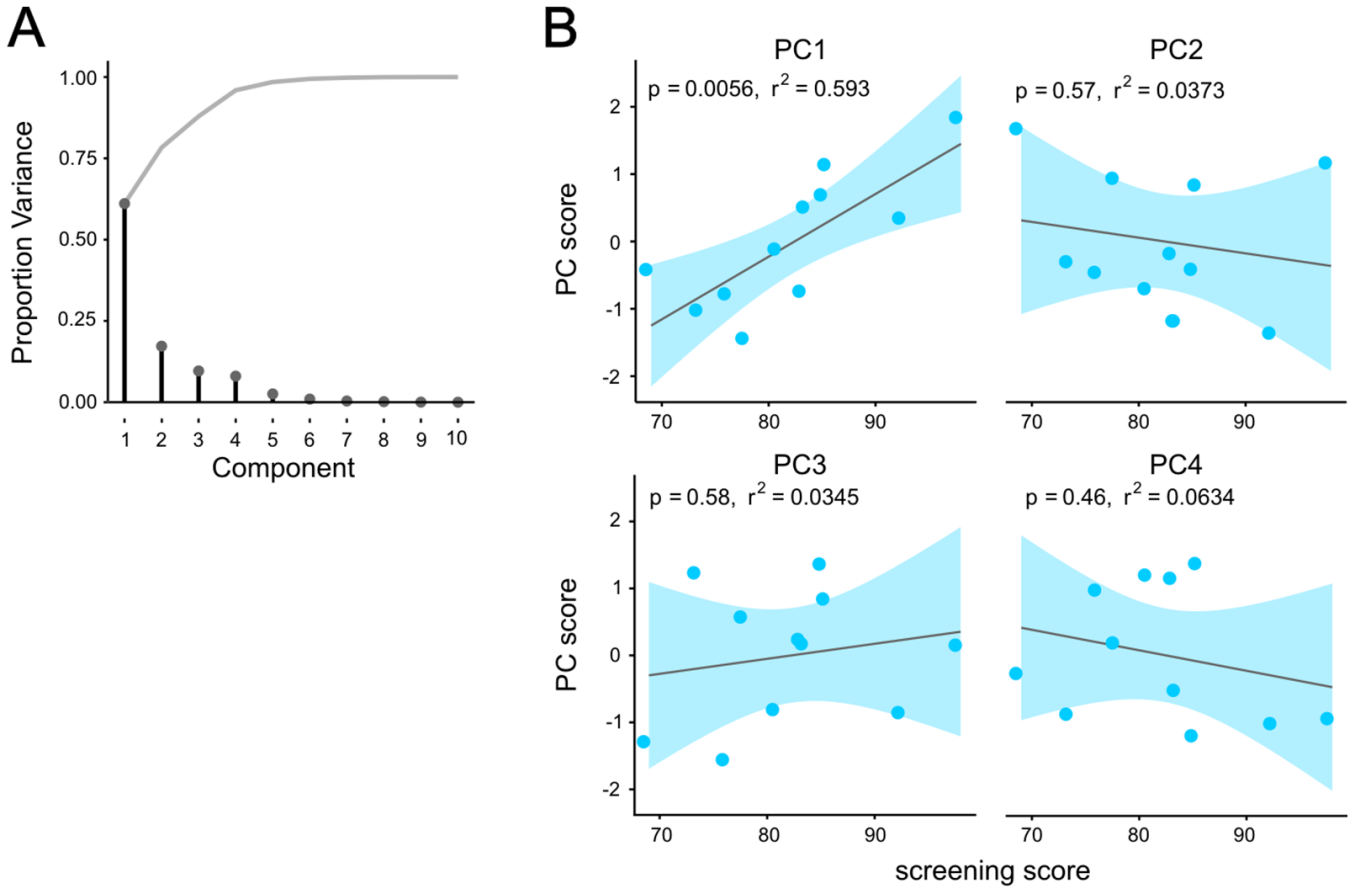
Relationship of 5-CSRTT LFP correlates to phenotypic impulsivity. A) Scree plot of the proportion of variance explained by principal components extracted from LFP variables derived from peri-event LFP variables (Table S15 in File S1). Grey line shows the cumulative proportion of variance explained. Four components were sufficient to explain >95% of variance in the LFP data. B) Scatter plots of the relationship between individual rat's impulsivity screening score and their score on principal components 1–4 for 11 rats meeting the criterion for high impulsivity. Grey line represents the least-squares regression line. The 95% confidence interval is shown by the shaded blue area.

We examined whether the relationship between the LFP variables and impulsivity in the HI group had a common structure using principal components analysis (Figure 6A). From the group of 11 rats, 10 LFP variables were extracted (Table S15 in File S1). One PC (PC1, accounting for 25% of variance) was significantly correlated with screening score (*P* = 0.006, r^2^ = 0.593, linear regression, Figure 6B), suggesting that in HI rats, impulsivity is a common factor which explains variance in a number of difference LFP windows. In particular, 3 variables which had positive loading greater than 0.4 on PC1 (post-poke gamma60 in the NAcb and PRL, and post-poke PAC in the PRL) were related to trial outcome, with a positive correlation with screening score and PC1, and positive loadings on the LFP variables indicating that in HI rats higher levels of impulsivity were associated with larger differences in LFP signals following correct and error responses (see Table S16 in File S1 for the loadings of each LFP variable on the 4 PCs, and Table S17 in File S1 for each rat's scores on each PC). A further LFP variable, the difference between theta power on previously rewarded and non-rewarded trials in LFPs recorded in NAcbC, had a negative loading on PC1. This suggests that in HI rats, higher levels of impulsivity may also be correlated with a reduced relationship between NAcbC theta oscillations and the outcome of past trials. We extracted the same LFP features from the 4 non-HI rats to test whether the non-HI rats showed the same relationship between PC1 and impulsivity (Figure S6B), but they did not (linear regression for all rats *P* = 0.140, r^2^ = 0.160). Importantly, these correlations were observed during recording sessions where the rats were required to wait 5 seconds before stimulus presentation, rather than the 7 second delay during impulsivity screening sessions. During the recording sessions, the HI rats did not make higher average numbers of premature responses compared to the non-HI rats (*F*_1,13_ = 0.038, *P* = 0.848), and there was no correlation between impulsivity screening score and average premature responses during recording sessions (either for all rats, *P* = 0.704, r^2^ = 0.012, or for only the HI sub-group *P* = 0.960, r^2^ = 0.000, Figure S6C), suggesting the relationship between LFP factors and screening score was not related simply to the number of premature responses performed in a session.

This analysis indicates that in HI rats, higher levels of impulsivity are associated with larger differences between correct and error LFP responses immediately following the completion of a nose-poke, but smaller LFP signals related to that outcome persisting into the next trial. However, this pattern does not appear to be true for rats which did not meet the criteria for “high impulsivity”. With only 4 rats not meeting the criteria for high impulsivity, and without a distinct “low impulsive” group specifically selected for very low levels of premature responding, as in previous published works, however, we cannot make any conclusions about whether, or how prior screening scores relates to LFP signals in non-HI rats.

## Discussion

We report several LFP correlates of waiting behaviour and reward anticipation and outcome in the PFC and NAcb of rats performing a behavioural task involving visual attention and action restraint. Our analysis demonstrates specific LFP events that take place at the onset of waiting, where pre-potent responses must be suppressed, as well as following responses leading to rewarding or non-rewarding outcomes, in gamma60 and theta frequency bands; and gamma-delta PAC. We show that theta and gamma60 power in the NAcb are significantly affected by upcoming impulsive behaviours, and that outcome-related LFP signals in NAcb and PRL correlate with trait-like impulsivity in a highly-impulsive subgroup of rats. Our findings thus support previous research showing a major involvement of medial PFC and NAcb in regulating specific subtypes of impulsivity [14], [21], [48].

### Gamma60 Oscillations and behaviour

We found a discrete gamma60 oscillation in both NAcb and PFC, changes in the power of which occurred during waiting and nose-poking, as well as correlating with both trial outcome and the outcome of previous trials. We found that following nose-poke responses gamma60 oscillations, particularly in the NAcb, differentiated between correct and error responses, in advance of reward receipt. A link between ventral striatal gamma60 oscillations and rewarding events has previously been reported [28], [29], [31], suggesting the outcome-related gamma60 activity we report might also be related to reward. However, other explanation of the post-response gamma60 data are possible, including (i) processing of reward or outcome related sensory cues (such as sounds associated with food pellet delivery or changes in box lighting which occurred shortly after correct nose-pokes); (ii) preparation for consummatory behaviours; (iii) switching attentional states (from waiting/attention to either obtaining and consuming food, or to error processing); (iv) in the case of error responses gamma60 increases might relate to regret or a counterfactual representation of the correct response or reward [84], [85]; (v) or some aspect of movement not accounted for by our analysis: for example, gamma oscillations in NAcb could be related to movement initiation or invigoration in certain motivational states, such as when animals are performing flexible approach behaviours, rather than cued behaviours, as has been suggested to drive single unit activity in NAcb [32]. In the 5-CSRTT while the rat's movement from nose-poking to the food magazine is self-motivated, it is invariant in distance and therefore we are not able to differentiate whether the gamma60 response to correct outcomes we observed was related to reward anticipation compared to the invigoration of movement based on reward proximity. However, given previous findings associating ventral striatal gamma oscillations [28], [29], [31] and single unit activity to reward anticipation [30], [86], [87], we believe this may be the most parsimonious explanation for the post-response gamma60 activity we observed.

Gamma60 oscillations also increased in power during the start of waiting, when rats typically engage in “scanning” behaviour [33], [46], [47]. Previously, it has been reported that neurons in the ventral striatum, which are activated by reward, are also activated during choice-points where rats must make a decision about which route to take to obtain reward [30]. Thus, transient increases in gamma60 power at the initiation of waiting could represent the potential reward that could be obtained by engaging in a trial. Further, we found that gamma60 power at this event was higher when the trial eventually ended in a premature response.

However, while LFP data alone provided informative predictions about upcoming behaviour (the LFP-only predictive model had an AUC of 0.6694), behavioural data (using rat's previous reward history and wait-start latency) provided more accurate predictions of upcoming responses, and these predictions could not be improved by adding LFP data. This suggests that the predictive LFP features discovered around the wait-start event may reflect task behavioural parameters, rather than containing additional information.

If the wait-start increase in gamma60 power was associated with the representation of possible reward, alterations in this reward signal would be in keeping with models of impulsive behaviour which suggest an association between impulsivity and alterations in brain reward systems [88]. For example, if a gamma60 representation of upcoming reward were larger on some trials, this might increase the motivation to respond, leading to failures of action restraint and increased impulsive behaviours.

We also found that in addition to a potential relationship to reward, PFC and NAcb gamma60 power also increased following errors, and that these error-related responses were similar for different types of error. Thus, both premature errors, representing failures of action restraint, and incorrect errors, representing attentional failures resulted in a sustained increase in gamma60 power following nose-poking. If gamma60 oscillations in the NAcb are related to reward, the sustained nature of this representation could contribute to an error correction process or a counterfactual representation of the reward that could have been obtained from a correct response, as has been recently demonstrated to occur in ventral striatal single units [85]. Alternately, given that LFP oscillations represent summed local network activity and post-synaptic currents it could be the case that correct response and error –related gamma60 oscillations are generated by different sets of synaptic inputs and/or different ensembles of local neurons which are active during the post-response epoch, but which produce similar responses at the LFP level.

### Theta oscillations and behaviour

Similar to gamma60 oscillations, changes in PFC and NAcb theta power was associated with waiting and trial outcome, as well as previous trial outcomes. We observed increased theta power in the PFC and NAcb during waiting. Similar findings have been reported in maze tasks where theta power within and coherence between the hippocampus and frontal cortex or striatum has been shown to increase during epochs of working memory or attention [83], [89], [90], suggesting that in our task the waiting-related theta increase might also relate to the engagement of attention. Supporting this idea, we also found that following correct responses theta power remained elevated until rats returned to the food magazine to receive food reward, whereas following error responses theta power decreased immediately after nose-poking. If the increase in theta power we observed during waiting relates to attention, it is logical that the increased theta power would persist until reward was obtained on correct trials, and would drop immediately after error nose-pokes, as we observed.

### Relationship to previous studies

As well as increased theta power, the waiting period was bracketed by increases in gamma60 power. In a 3-choice variant of the 5-CSRTT, changes in PFC LFP phase-locking and single unit activity have been observed, as well as ramping activity in ventral tegmental area neurons [26], [27], [91]. As this response preceded nose-poke responses, transient increases in striatal and cortical DA may be responsible for the increased gamma60 power observed in the present study. Interestingly, sustained error-related signals of the same time-course as the observed error-related PAC have been reported in single-unit activity in the PFC [26]. This suggests that errors could be represented in the PFC at multiple levels: changes in the activity of individual neurons, associated with changes in LFP oscillations, and their interactions. However, in contrast to the results reported for the 3-choice variant of the task above, we did not observe significantly lower theta power in the PRL, or indeed any brain region, preceding incorrect nose-pokes compared to correct responses [27]. Similarly, we did not find a decrease in theta power before a premature response; rather we found that theta power, particularly in the medial PFC increased before a premature response, an effect which interacted with the outcome of previous trials (theta power during waiting was particularly elevated before a premature response when rats were previously rewarded). Increased theta power during trials ending in premature responses might relate to some form of compensatory signal related to attention or action restraint. Alternatively if changes in theta power were related to a representation of past or upcoming rewards, this reward-related signal might be directly related to impulsivity [88].

However, these potential explanations do not explain our observed increased theta power compared to the previously observed decrease in theta power preceding incorrect and premature responses [27]. These discrepancies may be due to procedural differences. For example, rats in the present study were required to wait for a shorter period (5 versus 8 seconds) and the stimulus duration was longer (0.5 versus 0.3 seconds). Thus, our version of the task may have been less demanding in terms of response inhibition and attentional demand.

We observed that changes in both gamma60 and theta oscillations were correlated with the outcome of the previous trial, similar to reports of single unit activity in the ventral striatum [92]–[95]. With respect to gamma60, encoding of previous outcome appeared to be most strongly linked to the increase in gamma60 power at the start of waiting, whereas previous outcome was found to significantly influence theta power during the entire waiting period and persisted after nose-poking, thereby allowing signals related to previous trial outcome and upcoming response outcome to co-occur. Sustained representations of previous outcomes in the gamma60 and theta bands have been previously reported [29], [96] and may represent a neurobiological substrate for the production of adaptive behaviours based on the outcome of previous actions. Furthermore, one LFP variable associated with impulsivity in the highly impulsivity group of rats involved reward history, suggesting that impulsive rats may exhibit deficits in LFP signals related to previous rewards.

It has been previously suggested that there is a correlation between gamma60 power and movement, although the strength of this relationship has varied in different studies [29], [31], [97]. In the current experiment we found only limited evidence for a correlation between movement and gamma60 or theta power during 5-CSRTT performance. One suggested correlate of gamma oscillations in the NAcb has been times of movement initiation [97]. In the 5-CSRTT the events where the rat left the food magazine or made a nose-poke were fixed times of movement initiation, but we found no evidence that velocity affected either gamma60 or theta power at these times. However, our task was performed in an operant chamber, compared to previous studies using maze-based tasks. Therefore the movements in our task were mostly rotational or extremely short in duration and displacement, compared to longer maze runs, which could explain differences in LFP-movement relationships. Further, our recordings were from more rostral areas of the NAcb, which may have different anatomical inputs [15], [16], [98]–[103] and therefore different functions to the more caudal and lateral regions of the NAcb studied in previous reports. Alternatively our data may support the argument that in general NAcb gamma oscillations are not well correlated with movement.

It is possible that the LFP oscillations we observed could have arisen from volume conduction from a source distant to the NAcb or PFC: for example the nearby piriform cortex, where gamma oscillations are prominent [28], [104]. Volume conduction from a distant source could also plausibly explain the regional differences in LFP power we observed if LFP volume conduction varied along a spatial gradient.

However, we believe volume conduction is unlikely to be the sole explanation for our findings. Firstly, following re-referencing LFPs by subtracting the mean of all simultaneously recorded signals in the same structure, which might be expected to remove common signal components (such as those arising from volume conduction), we still observed peaks in power spectra for theta and gamma60 oscillations.

Second, previous reports indicate that the striatum has the properties required for the generation of LFPs. In recordings from anaesthetised rats and slice preparations, oscillations in striatal cell membrane potentials have been observed, which accompany oscillations in the LFP [105]–[107]. Changes in the extracellular field potential have also been observed in slice preparations in response to electrical stimulation [108], [109], indicating that isolated sections of striatum are capable of producing a LFP.

These findings have also been supported in awake, behaving rats. Complete reversal of the phase of high-voltage spindles has been observed across striatum [110], as would be expected if these oscillations were locally generated (by contrast volume-conducted oscillations would have constant phase), and the theta oscillation recorded in dorsomedial striatum has been shown to remain following re-referencing [78] and to not consistently correlate with hippocampal theta oscillations [90]). Gamma frequency activity within the ventral striatum has been shown to be heterogeneous [31] (which would not be expected if gamma oscillations were conducted from a distant structure), and simultaneously recorded single units exhibit phase-locking to theta and gamma oscillations [28], [29], [31], [111], [112]. Similarly, single units recorded in rat medial PFC have also been reported to phase-lock to delta and theta oscillations [27], [89], [113], [114], and the degree of this phase-locking varies between correct and error responses, and during reward consumption in behavioural tasks.

We therefore conclude that it is most likely, based on our re-referencing analysis, and previous experimental data, that the LFP oscillations we recorded were generated locally in the PFC and NAcb, although there remains a possibility that volume conduction also contributed to the observed results.

### Behaviour-related phase-amplitude coupling

We show that gamma60 oscillations in NAcb and medial PFC are coupled to 2–4 Hz oscillations (Figure 5), and are significantly weaker in PRL compared with the IL and NAcb. Our findings of gamma-delta PAC in the rat are consistent with previous studies. Gamma oscillations in the rat PFC have been associated with a 2–4 Hz delta oscillation [83], similar to NAcb and PRL gamma60 oscillations in the mouse [82]. By contrast, in rat orbitofrontal cortex, gamma oscillations have been associated with theta oscillations [68], and in rat dorsal striatum high-gamma (80 Hz) oscillations also appear to couple to theta oscillations [80]. In humans, NAcb gamma oscillations have been shown to couple to 12 Hz alpha oscillations [79]. These data suggest that a general feature of corticostriatal gamma oscillations is coupling to low frequency oscillations, and in the rat more medial structures have a stronger relationship with delta, and more lateral structures, with theta. These distinctions could relate to anatomical differences between the various regions, with delta coupling relating to input from midbrain DA neurons [83].

In contrast to gamma60 power, gamma60-delta PAC showed no clear correlation with the initiation of waiting, but did differentiate correct and error responses following nose-poking and the receipt of outcome-related cues. However, unlike the effect of outcome on gamma60 power, outcome-related changes in PAC emerged later, and lacked the transient correct response-related increase in power. Therefore, information encoded by gamma60-delta PAC appears to differ from that encoded by gamma60 power alone. This might suggest gamma60 oscillations only align to lower frequency oscillations during particular behavioural states, perhaps related to different structures providing input to the NAcb [115], [116].

### LFP correlates of phenotypic impulsivity

From the large number of LFP correlates of 5-CSRTT performance we identified a subgroup which correlated with rat's impulsivity screening scores, in a subset of rats classed as “highly-impulsive”, and which contributed to a principal component correlated with impulsivity screening score. Importantly, these task-related correlates of screening were derived from 5-CSRTT recording sessions with 5 second waiting period where HI rats did not make increased numbers of premature responses relative to their non-highly impulsive counterparts, and where there was no correlation between premature responding and screening score. Therefore our findings were not simply explained by an increased frequency of premature responding in HI rats.

The LFP factor correlating with impulsivity screening score in highly impulsive rats had large positive loadings on post-response signals of trial outcome in gamma60 oscillations in the NAcbC and PRL, as well as in PAC in the PRL, accompanied by negative loadings on signals related to previous trial outcome in theta oscillations in the NAcbC during waiting. This suggests that in phenotypically highly-impulsive animals, higher levels of impulsivity during screening sessions were associated with increased representations of the outcome of behaviour occurring immediately after the rats performed a nose-poke, and received information about its outcome, but also with reductions in those signals during subsequent behaviours, where information about the outcome of past behaviours might be important in shaping behaviour. Thus, highly impulsive animals appear to have alterations in LFP signals related to the outcome behaviour, which could explain why, when challenged with increased waiting demands, as required during the impulsivity screening sessions, they make persistently high numbers of impulsive responses.

Our findings may be relevant to clinical disorders of impulsivity such as ADHD where altered reinforcement mechanisms are implicated. Individuals diagnosed with ADHD are postulated to require stronger, more salient stimuli to control behaviour, and are sensitive to delayed rewards where subjective value is sharply diminished [88]. The findings of the present study are consistent with this hypothesis by suggesting that the inability to suppress anticipatory responses for future rewards may be determined by deficits in encoding recent reward history.

## Supporting Information

Figure S1 **Whole-session Power Spectral Density and re-referencing.** A) Power Spectral Density (PSD) for all electrodes located in NAcbC, NAcbSh, PRL or IL, calculated from z-scored raw data over 30 minute recording sessions. Solid line shows the mean of all trials. The shaded area shows the SEM. B) PSD calculated from data after re-referencing by subtracting the average signal of all simultaneously recorded electrodes in the same brain region, and then z-scoring the resultant signal. Non re-referenced PSDs are shown in grey for comparison. Solid line shows the mean. The shaded area shows the SEM.
(TIF)

Figure S2 **Windowed gamma60 and theta LFP power around wait-start.** A) Average gamma60 power from 0.25 seconds before to 0.25 seconds after wait-start (bar charts show mean power and 95% confidence interval (from normal distribution)). B) Bar charts of average theta power from 0.75 seconds to 2 seconds after wait-start.
(TIF)

Figure S3 **Windowed gamma60 and theta LFP power around nose-poke responding.** A) Average gamma60 power from 0.25 seconds before to the time of nose-poking. B) Average gamma60 power from 0.25 seconds to 2 seconds after nose-poking. C) Average theta power from 1 second before to the time of nose-poking. D) Average theta power from 0.25 seconds to 0.9 seconds after nose-poking. E) Average theta power from 1 second to 2 seconds after nose-poking.
(TIF)

Figure S4 **Nose-poke responding LFP power binned by magazine latency.** A) Peri nose-poke z-scored gamma60 power, binned by magazine return latency. As magazine return latencies were much faster for correct trials compared to error trials, trials are binned by the log10 of the magazine return latency to improve plot interpretability. Bins with no trials are horizontal solid blue. The vertical white line represents the time of nose-poking, vertical white lines within each row are the magazine return latency in that bin. As in Figure 3, trials are divided by brain region, task outcome and previous reward. B) As A, plotting z-scored theta power.
(TIF)

Figure S5 **Gamma60-delta phase amplitude coupling.** A) Phase-amplitude coupling in PFC and NAcb calculated over whole 30 minute recordings. PAC was calculated between pairs of electrodes recorded simultaneously in the same structure, with one electrode giving amplitude data, and the other giving phase data. PAC was calculated for all possible pairs of electrodes and averaged to give a single PAC value per session. B) Average gamma60-delta phase-amplitude coupling (PAC) from 1 second before to the time of wait-start. C) Average gamma60-delta PAC from 0.75 seconds to 2 seconds after nose-poking.
(TIF)

Figure S6 **Correlation between 5-CSRTT LFPs and impulsivity score.** A) Scatter plots showing the correlation between 10 LFP variables extracted from the peri-event windows (Table S15 in File S1) and impulsivity screening scores for all rats. Rats meeting the criterion for high impulsivity are shown in blue; rats not meeting this criterion are shown in orange. Lines present the least squares regression line and its 95% confidence interval. Black text gives regression data for all rats, blue text regression data for highly impulsive rats only. As only 4 rats were not highly-impulsive, they were not analysed. B) Scatter plots showing the correlation between 4 scores on 4 Principal components and impulsivity screening scores. Lines and text as A. C) Scatter plot showing the correlation between impulsivity screening scores, and the average number of premature responses performed on 5-CSRTT sessions for all rats. Lines and text as A.
(TIF)

File S1 Table S1, Electrode placements in all rats. Table S2, Wait-start latencies for all rats. Table S3, Total unique trials analysed (in column headings (+): previously rewarded trials, (−): previously non-rewarded trials). Table S4, Total trials recorded by all electrodes. Table S5, Wait-start gamma60 power [−0.25 0.25]. Table S6, Wait-start theta power [0.75 2]. Table S7, Nose-poke gamma60 power [−0.25 0]. Table S8, Nose-poke gamma60 power [0.25 2]. Table S9, Nose-poke theta power [−1 0]. Table S10, Nose-poke theta power [0.25 0.9]. Table S11, Nose-poke theta power [1 2]. Table S12, Wait-start gamma60-delta PAC [−1 0]. Table S13, Nose-poke PAC [0.75 2]. Table S14, Impulsivity Screening Scores. Table S15, LFP variables. Table S16, PC loadings on LFP variables. Table S17, Factor scores for all rats.
(DOCX)
