# Supplementary figures and images for: Oscillatory Activity in the Medial Prefrontal Cortex and Nucleus Accumbens Correlates with Impulsivity and Reward Outcome
Source: PLoS One. 2014 Oct 21;9(10):e111300. doi: 10.1371/journal.pone.0111300 (PMC4205097; doi:10.1371/journal.pone.0111300)

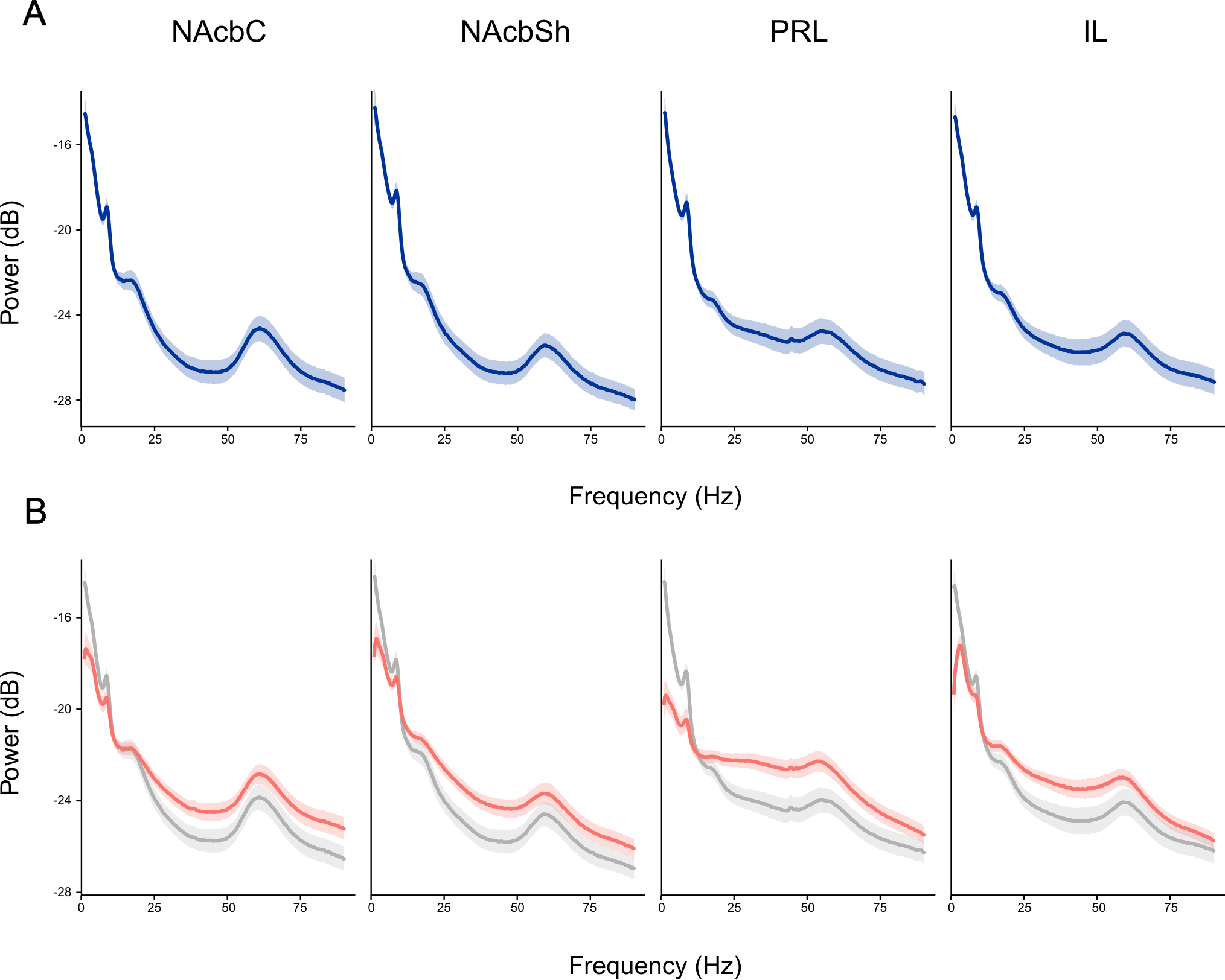

Supplement: Figure S1 — Whole-session Power Spectral Density and re-referencing. A) Power Spectral Density (PSD) for all electrodes located in NAcbC, NAcbSh, PRL or IL, calculated from z-scored raw data over 30 minute recording sessions. Solid line shows the mean of all trials. The shaded area shows the SEM. B) PSD calculated from data after re-referencing by subtracting the average signal of all simultaneously recorded electrodes in the same brain region, and then z-scoring the resultant signal. Non re-referenced PSDs are shown in grey for comparison. Solid line shows the mean. The shaded area shows the SEM. (TIF) [file pone.0111300.s001.tif]

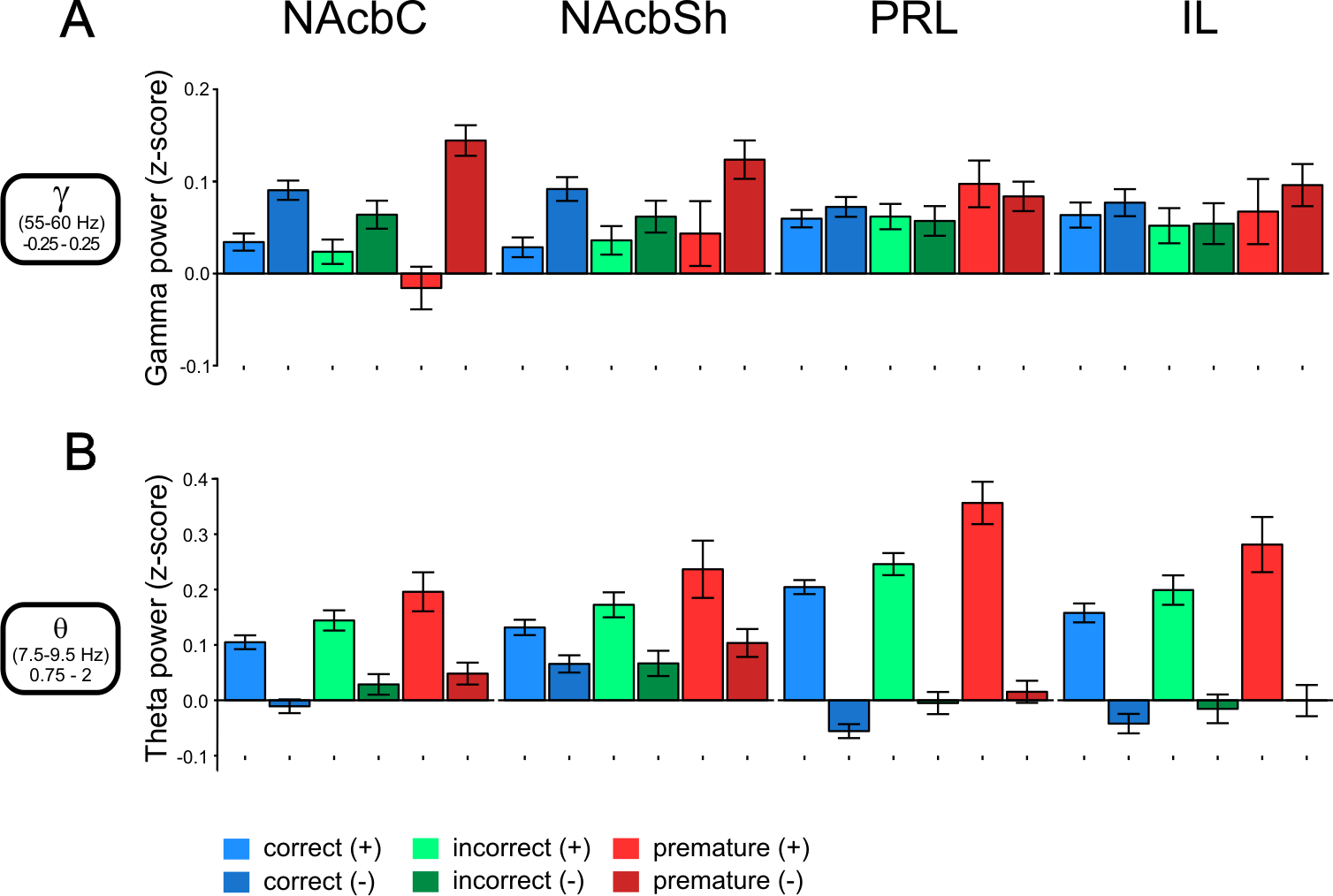

Supplement: Figure S2 — Windowed gamma60 and theta LFP power around wait-start. A) Average gamma60 power from 0.25 seconds before to 0.25 seconds after wait-start (bar charts show mean power and 95% confidence interval (from normal distribution)). B) Bar charts of average theta power from 0.75 seconds to 2 seconds after wait-start. (TIF) [file pone.0111300.s002.tif]

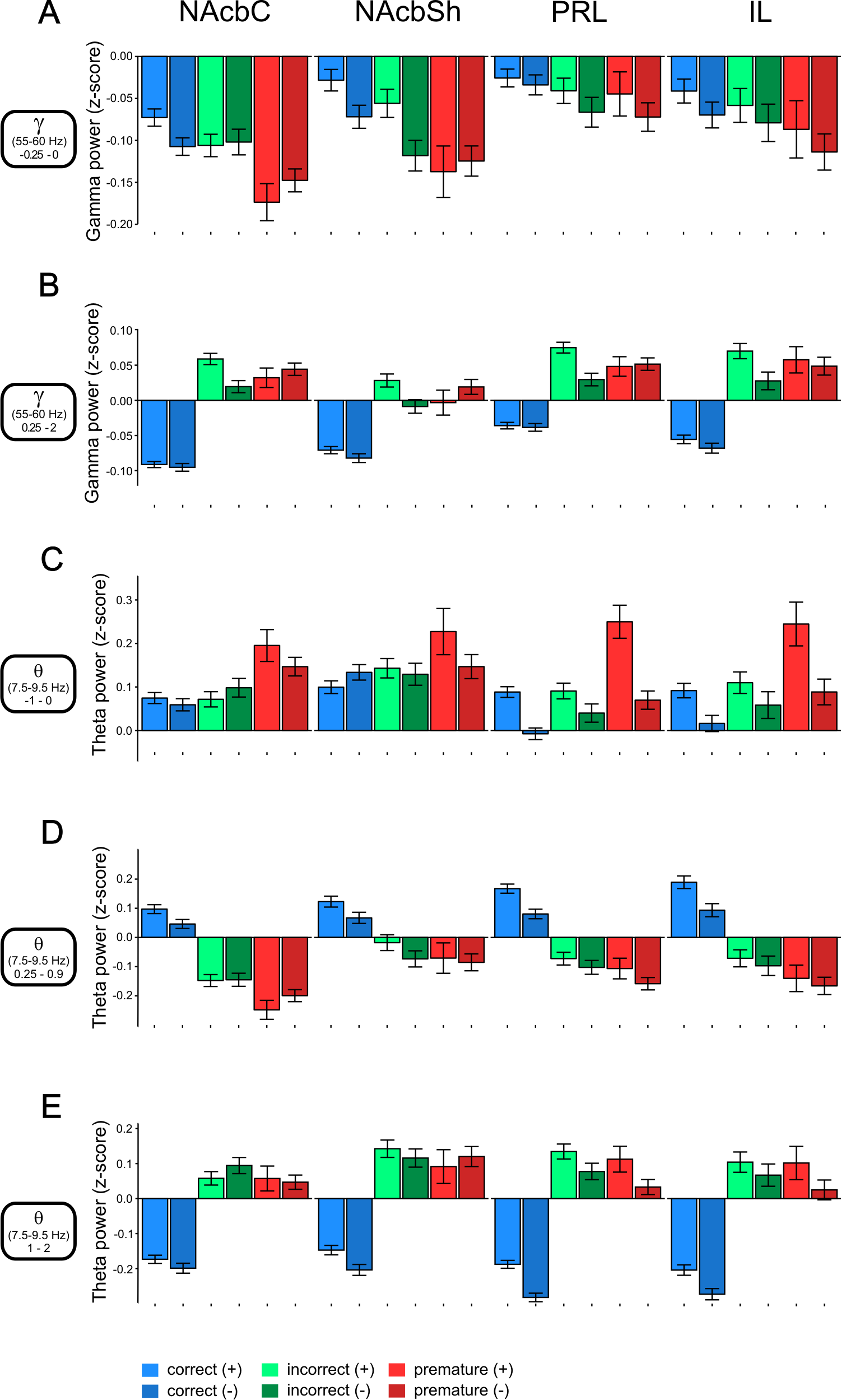

Supplement: Figure S3 — Windowed gamma60 and theta LFP power around nose-poke responding. A) Average gamma60 power from 0.25 seconds before to the time of nose-poking. B) Average gamma60 power from 0.25 seconds to 2 seconds after nose-poking. C) Average theta power from 1 second before to the time of nose-poking. D) Average theta power from 0.25 seconds to 0.9 seconds after nose-poking. E) Average theta power from 1 second to 2 seconds after nose-poking. (TIF) [file pone.0111300.s003.tif]

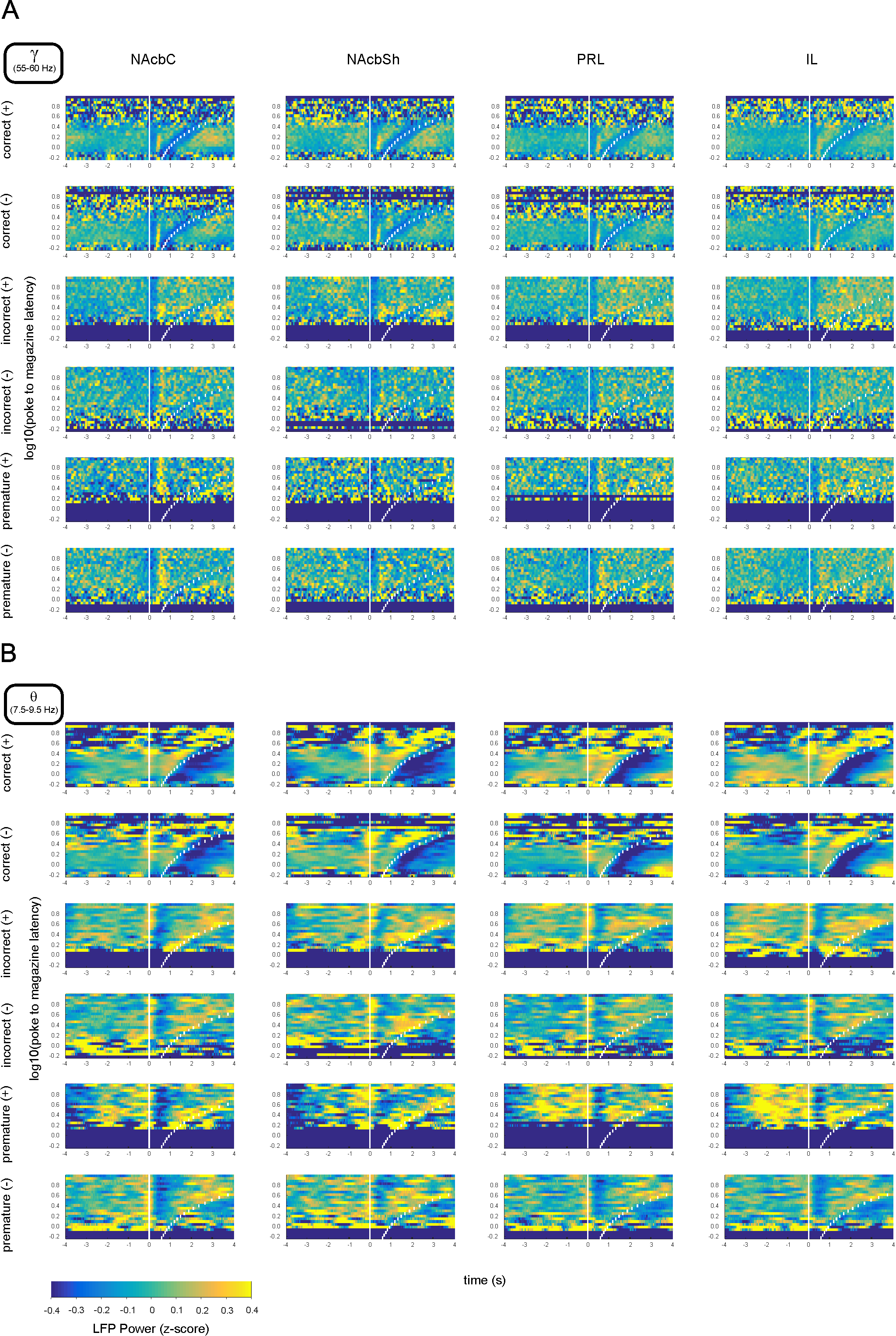

Supplement: Figure S4 — Nose-poke responding LFP power binned by magazine latency. A) Peri nose-poke z-scored gamma60 power, binned by magazine return latency. As magazine return latencies were much faster for correct trials compared to error trials, trials are binned by the log10 of the magazine return latency to improve plot interpretability. Bins with no trials are horizontal solid blue. The vertical white line represents the time of nose-poking, vertical white lines within each row are the magazine return latency in that bin. As in Figure 3, trials are divided by brain region, task outcome and previous reward. B) As A, plotting z-scored theta power. (TIF) [file pone.0111300.s004.tif]

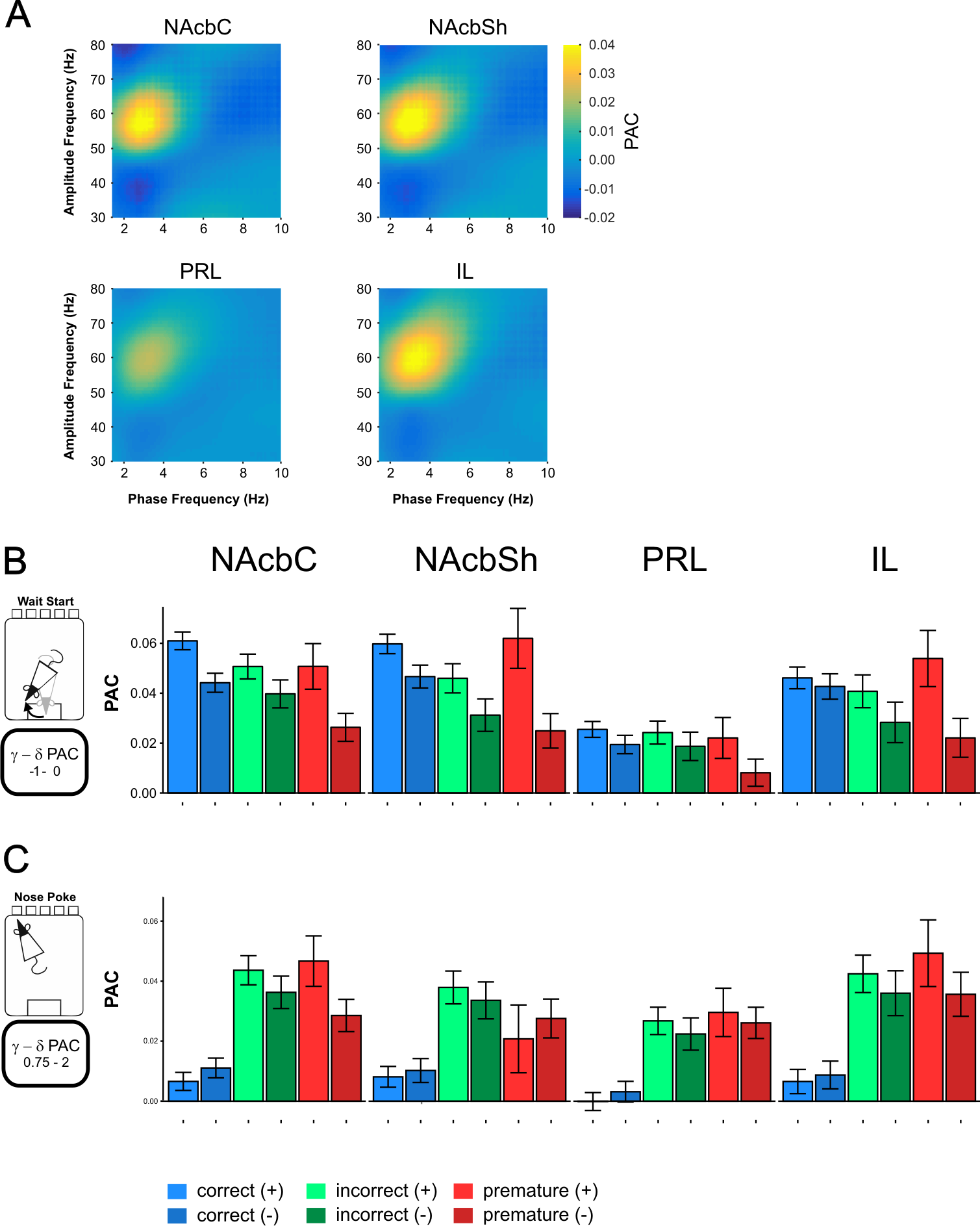

Supplement: Figure S5 — Gamma60-delta phase amplitude coupling. A) Phase-amplitude coupling in PFC and NAcb calculated over whole 30 minute recordings. PAC was calculated between pairs of electrodes recorded simultaneously in the same structure, with one electrode giving amplitude data, and the other giving phase data. PAC was calculated for all possible pairs of electrodes and averaged to give a single PAC value per session. B) Average gamma60-delta phase-amplitude coupling (PAC) from 1 second before to the time of wait-start. C) Average gamma60-delta PAC from 0.75 seconds to 2 seconds after nose-poking. (TIF) [file pone.0111300.s005.tif]

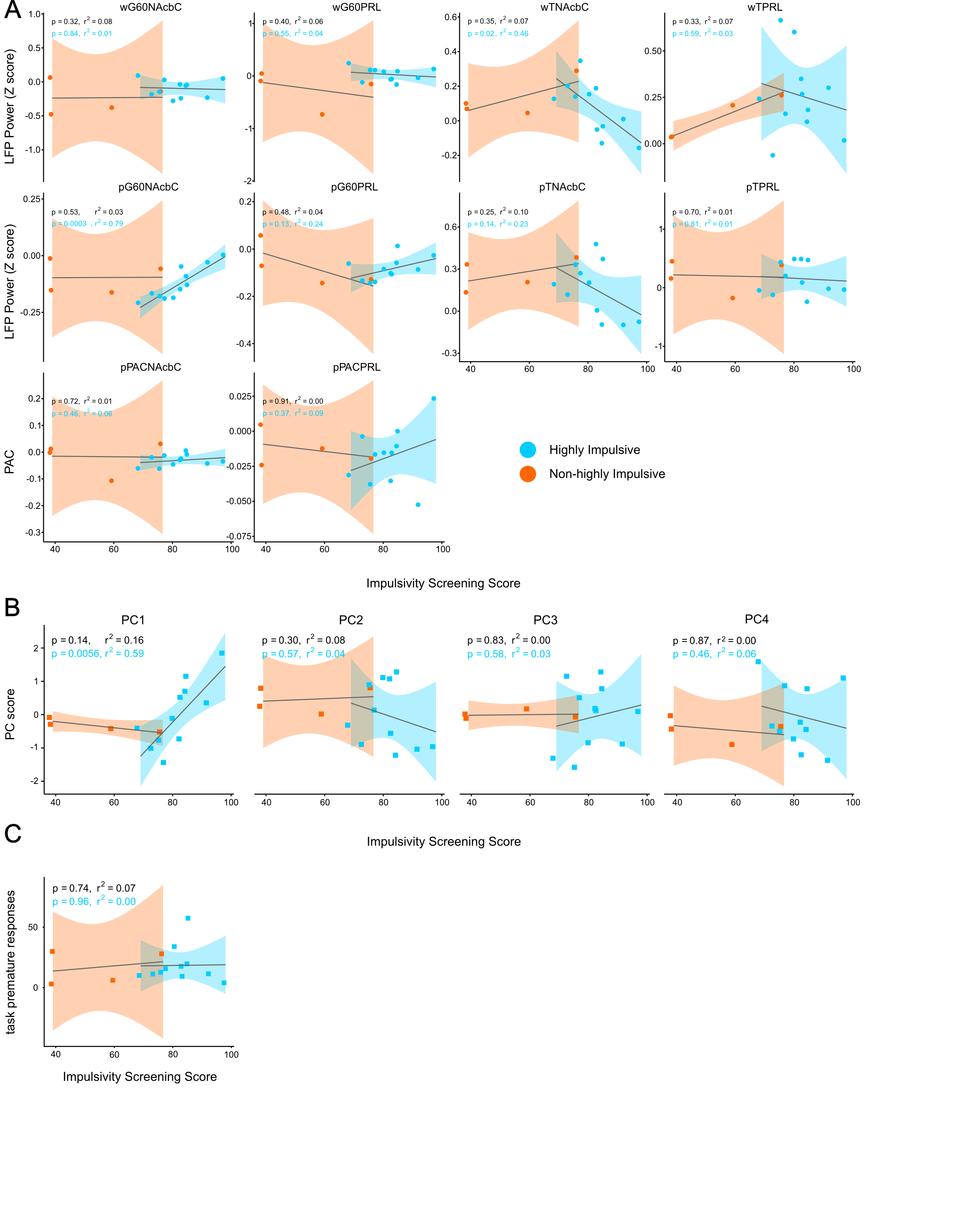

Supplement: Figure S6 — Correlation between 5-CSRTT LFPs and impulsivity score. A) Scatter plots showing the correlation between 10 LFP variables extracted from the peri-event windows (Table S15 in File S1) and impulsivity screening scores for all rats. Rats meeting the criterion for high impulsivity are shown in blue; rats not meeting this criterion are shown in orange. Lines present the least squares regression line and its 95% confidence interval. Black text gives regression data for all rats, blue text regression data for highly impulsive rats only. As only 4 rats were not highly-impulsive, they were not analysed. B) Scatter plots showing the correlation between 4 scores on 4 Principal components and impulsivity screening scores. Lines and text as A. C) Scatter plot showing the correlation between impulsivity screening scores, and the average number of premature responses performed on 5-CSRTT sessions for all rats. Lines and text as A. (TIF) [file pone.0111300.s006.tif]
