# Supplementary material for: Oscillatory Activity in the Medial Prefrontal Cortex and Nucleus Accumbens Correlates with Impulsivity and Reward Outcome
Source: PLoS One. 2014 Oct 21;9(10):e111300. doi: 10.1371/journal.pone.0111300 (PMC4205097; doi:10.1371/journal.pone.0111300)
Supplement: File S1 — Table S1, Electrode placements in all rats. Table S2, Wait-start latencies for all rats. Table S3, Total unique trials analysed (in column headings (+): previously rewarded trials, (−): previously non-rewarded trials). Table S4, Total trials recorded by all electrodes. Table S5, Wait-start gamma60 power [−0.25 0.25]. Table S6, Wait-start theta power [0.75 2]. Table S7, Nose-poke gamma60 power [−0.25 0]. Table S8, Nose-poke gamma60 power [0.25 2]. Table S9, Nose-poke theta power [−1 0]. Table S10, Nose-poke theta power [0.25 0.9]. Table S11, Nose-poke theta power [1 2]. Table S12, Wait-start gamma60-delta PAC [−1 0]. Table S13, Nose-poke PAC [0.75 2]. Table S14, Impulsivity Screening Scores. Table S15, LFP variables. Table S16, PC loadings on LFP variables. Table S17, Factor scores for all rats. (DOCX) [file pone.0111300.s007.docx]

## PLOS ONE Manuscript Supporting Information Tables

## *Supporting Table 1: Electrode placements for all rats*

| **Rat ID** | **NAcbC** | **NAcbS** | **PRL** | **IL** |
| --- | --- | --- | --- | --- |
| 101_44 | *8* | *8* | *6* | *4* |
| 102_54 | *3* | *7* | *6* | *6* |
| 158_02 | *7* | *7* | *6* | *2* |
| 158_08 | *1* | *5* | *4* | *0* |
| 158_30 | *7* | *8* | *6* | *4* |
| 158_34 | *6* | *4* | *5* | *4* |
| 158_40 | *0* | *6* | *6* | *4* |
| 158_47 | *9* | *3* | *6* | *4* |
| 229_11 | *9* | *2* | *6* | *4* |
| 229_17 | *9* | *1* | *6* | *4* |
| 229_22 | *6* | *6* | *6* | *4* |
| 229_23 | *9* | *0* | *6* | *4* |
| 229_30 | *3* | *7* | *6* | *4* |
| 229_45 | *0* | *0* | *4* | *0* |
| 94_03 | *7* | *8* | *8* | *2* |
| 97_14 | *8* | *2* | *8* | *4* |
| 97_16 | *9* | *2* | *4* | *0* |
| Total | *101* | *76* | *99* | *54* |

## *Supporting Table 2: Wait-Start Latencies*

| **Rat ID** | **Correct (+)** | **Correct (-)** | **Incorrect (+)** | **Incorrect (-)** | **Premature (+)** | **Premature (-)** |
| --- | --- | --- | --- | --- | --- | --- |
| 101_44 | 2.623844 | 1.832903 | 2.582767 | 1.727242 | 2.205495 | 1.248864 |
| 102_54 | 1.958454 | 0.997297 | 2.157471 | 0.964479 | 2.001052 | 0.787483 |
| 158_02 | 3.370119 | 2.043593 | 3.589463 | 2.270113 | 2.509583 | 1.349744 |
| 158_08 | 1.872814 | 1.426914 | 2.082426 | 2.046757 | 1.56372 | 1.228955 |
| 158_30 | 4.641516 | 1.992818 | 5.711354 | 1.878599 | 2.502764 | 1.205932 |
| 158_34 | 2.622569 | 2.039685 | 2.551364 | 2.286842 | 2.205856 | 1.233822 |
| 158_40 | 3.073984 | 1.082601 | 3.510549 | 1.112336 | 2.386754 | 0.919513 |
| 158_47 | 2.474886 | 1.783025 | 2.465612 | 2.248155 | 1.917911 | 1.137588 |
| 229_11 | 1.742563 | 0.67304 | 1.788174 | 0.736169 | 1.837839 | 0.745944 |
| 229_17 | 2.110516 | 1.524058 | 1.94662 | 1.345498 | 1.694039 | 1.221373 |
| 229_22 | 2.025658 | 1.931833 | 1.993405 | 1.721477 | 1.795299 | 1.118089 |
| 229_23 | 2.191841 | 1.753765 | 2.401296 | 1.634707 | 1.632303 | 1.214145 |
| 229_30 | 2.911875 | 2.367947 | 2.865639 | 2.436366 | 2.207496 | 1.407918 |
| 229_45 | 1.995914 | 1.674517 | 2.07469 | 1.629117 | 1.473466 | 1.224025 |
| 94_03 | 3.551537 | 2.015599 | 3.94991 | 2.021287 | 2.827654 | 1.238174 |
| 97_14 | 3.3427 | 1.318988 | 3.415419 | 1.217897 | 2.250239 | 0.765572 |
| 97_16 | 2.767816 | 1.614135 | 2.795903 | 1.425723 | 2.925237 | 1.465384 |
| Overall (mean of all trials pooled) | 2.485514 | 1.695775 | 2.763166 | 1.721815 | 1.984702 | 1.191002 |
| SEM (SD of all trials/sqrt(n rats)) | 0.2389092 | 0.2052806 | 0.3281711 | 0.2401928 | 0.1609960 | 0.1374206 |

## *Supporting Table 3: Total unique trials*

| **Rat ID** | **Correct (+)** | **Correct (-)** | **Incorrect (+)** | **Incorrect (-)** | **Premature (+)** | **Premature (-)** | **Total** | **Total recording Sessions** |
| --- | --- | --- | --- | --- | --- | --- | --- | --- |
| 101_44 | 85 | 56 | 36 | 14 | 13 | 7 | 211 | 2 |
| 102_54 | 52 | 20 | 14 | 5 | 3 | 5 | 99 | 1 |
| 158_02 | 43 | 78 | 37 | 32 | 33 | 70 | 293 | 3 |
| 158_08 | 137 | 79 | 66 | 38 | 7 | 8 | 335 | 4 |
| 158_30 | 29 | 152 | 44 | 107 | 6 | 134 | 472 | 6 |
| 158_34 | 63 | 43 | 34 | 23 | 9 | 41 | 213 | 2 |
| 158_40 | 230 | 118 | 89 | 29 | 14 | 17 | 497 | 5 |
| 158_47 | 164 | 99 | 64 | 28 | 23 | 50 | 428 | 4 |
| 229_11 | 166 | 146 | 87 | 62 | 27 | 47 | 535 | 7 |
| 229_17 | 207 | 195 | 93 | 69 | 85 | 97 | 746 | 7 |
| 229_22 | 413 | 162 | 124 | 72 | 16 | 57 | 844 | 9 |
| 229_23 | 183 | 172 | 102 | 58 | 22 | 84 | 621 | 7 |
| 229_30 | 269 | 161 | 97 | 41 | 37 | 32 | 637 | 7 |
| 229_45 | 254 | 178 | 113 | 60 | 44 | 109 | 758 | 7 |
| 94_03 | 175 | 199 | 153 | 120 | 28 | 70 | 745 | 7 |
| 97_14 | 112 | 70 | 58 | 29 | 3 | 5 | 277 | 3 |
| 97_16 | 101 | 36 | 28 | 20 | 3 | 8 | 196 | 2 |
| Total | 2683 | 1964 | 1239 | 807 | 373 | 841 | 7907 | 83 |

## *Supporting Table 4: Total trials recorded over all electrodes*

| **Rat ID** | **Brain Region** | **Correct (+)** | **Correct (-)** | **Incorrect (+)** | **Incorrect (-)** | **Premature (+)** | **Premature (-)** | **Total** |
| --- | --- | --- | --- | --- | --- | --- | --- | --- |
| 101_44 | NAcbC | 588 | 392 | 252 | 98 | 91 | 49 | 1470 |
|  | NAcbSh | 672 | 448 | 288 | 112 | 104 | 56 | 1680 |
|  | PRL | 420 | 280 | 180 | 70 | 65 | 35 | 1050 |
|  | IL | 336 | 224 | 144 | 56 | 52 | 28 | 840 |
|  | Total | 2016 | 1344 | 864 | 336 | 312 | 168 | 5040 |
| 102_54 | NAcbC | 156 | 60 | 42 | 15 | 9 | 21 | 303 |
|  | NAcbSh | 364 | 140 | 98 | 35 | 21 | 49 | 707 |
|  | PRL | 312 | 120 | 84 | 30 | 18 | 42 | 606 |
|  | IL | 312 | 120 | 84 | 30 | 18 | 42 | 606 |
|  | Total | 1144 | 440 | 308 | 110 | 66 | 154 | 2222 |
| 158_02 | NAcbC | 129 | 234 | 111 | 96 | 99 | 210 | 879 |
|  | NAcbSh | 172 | 312 | 148 | 128 | 132 | 280 | 1172 |
|  | PRL | 86 | 156 | 74 | 64 | 66 | 140 | 586 |
|  | IL | 0 | 0 | 0 | 0 | 0 | 0 | 0 |
|  | Total | 387 | 702 | 333 | 288 | 297 | 630 | 2637 |
| 158_08 | NAcbC | 136 | 79 | 66 | 38 | 7 | 8 | 334 |
|  | NAcbSh | 680 | 395 | 330 | 190 | 35 | 40 | 1670 |
|  | PRL | 408 | 237 | 198 | 114 | 21 | 24 | 1002 |
|  | IL | 0 | 0 | 0 | 0 | 0 | 0 | 0 |
|  | Total | 1224 | 711 | 594 | 342 | 63 | 72 | 3006 |
| 158_30 | NAcbC | 203 | 1064 | 308 | 749 | 42 | 938 | 3304 |
|  | NAcbSh | 232 | 1216 | 352 | 856 | 48 | 1072 | 3776 |
|  | PRL | 174 | 912 | 264 | 642 | 36 | 804 | 2832 |
|  | IL | 87 | 456 | 132 | 321 | 18 | 402 | 1416 |
|  | Total | 696 | 3648 | 1056 | 2568 | 144 | 3216 | 11328 |
| 158_34 | NAcbC | 378 | 252 | 204 | 138 | 54 | 240 | 1266 |
|  | NAcbSh | 0 | 0 | 0 | 0 | 0 | 0 | 0 |
|  | PRL | 315 | 210 | 170 | 115 | 45 | 200 | 1055 |
|  | IL | 252 | 168 | 136 | 92 | 36 | 160 | 844 |
|  | Total | 945 | 630 | 510 | 345 | 135 | 600 | 3165 |
| 158_40 | NAcbC | 0 | 0 | 0 | 0 | 0 | 0 | 0 |
|  | NAcbSh | 460 | 236 | 178 | 58 | 28 | 34 | 994 |
|  | PRL | 460 | 236 | 178 | 58 | 28 | 34 | 994 |
|  | IL | 169 | 118 | 77 | 29 | 9 | 17 | 419 |
|  | Total | 1089 | 590 | 433 | 145 | 65 | 85 | 2407 |
| 158_47 | NAcbC | 330 | 198 | 128 | 56 | 44 | 100 | 856 |
|  | NAcbSh | 0 | 0 | 0 | 0 | 0 | 0 | 0 |
|  | PRL | 495 | 297 | 192 | 84 | 66 | 150 | 1284 |
|  | IL | 165 | 99 | 64 | 28 | 22 | 50 | 428 |
|  | Total | 990 | 594 | 384 | 168 | 132 | 300 | 2568 |
| 229_11 | NAcbC | 1485 | 1305 | 783 | 558 | 243 | 432 | 4806 |
|  | NAcbSh | 330 | 290 | 174 | 124 | 54 | 96 | 1068 |
|  | PRL | 990 | 870 | 522 | 372 | 162 | 288 | 3204 |
|  | IL | 660 | 580 | 348 | 248 | 108 | 192 | 2136 |
|  | Total | 3465 | 3045 | 1827 | 1302 | 567 | 1008 | 11214 |
| 229_17 | NAcbC | 1248 | 1170 | 552 | 414 | 516 | 576 | 4476 |
|  | NAcbSh | 0 | 0 | 0 | 0 | 0 | 0 | 0 |
|  | PRL | 1248 | 1170 | 552 | 414 | 516 | 576 | 4476 |
|  | IL | 832 | 780 | 368 | 276 | 344 | 384 | 2984 |
|  | Total | 3328 | 3120 | 1472 | 1104 | 1376 | 1536 | 11936 |
| 229_22 | NAcbC | 1648 | 644 | 496 | 288 | 64 | 232 | 3372 |
|  | NAcbSh | 2472 | 966 | 744 | 432 | 96 | 348 | 5058 |
|  | PRL | 2472 | 966 | 744 | 432 | 96 | 348 | 5058 |
|  | IL | 1236 | 483 | 372 | 216 | 48 | 174 | 2529 |
|  | Total | 7828 | 3059 | 2356 | 1368 | 304 | 1102 | 16017 |
| 229_23 | NAcbC | 1638 | 1539 | 909 | 522 | 198 | 774 | 5580 |
|  | NAcbSh | 0 | 0 | 0 | 0 | 0 | 0 | 0 |
|  | PRL | 1092 | 1026 | 606 | 348 | 132 | 516 | 3720 |
|  | IL | 728 | 684 | 404 | 232 | 88 | 344 | 2480 |
|  | Total | 3458 | 3249 | 1919 | 1102 | 418 | 1634 | 11780 |
| 229_30 | NAcbC | 804 | 483 | 291 | 120 | 111 | 99 | 1908 |
|  | NAcbSh | 1608 | 966 | 582 | 240 | 222 | 198 | 3816 |
|  | PRL | 804 | 483 | 291 | 120 | 111 | 99 | 1908 |
|  | IL | 804 | 483 | 291 | 120 | 111 | 99 | 1908 |
|  | Total | 4020 | 2415 | 1455 | 600 | 555 | 495 | 9540 |
| 229_45 | NAcbC | 0 | 0 | 0 | 0 | 0 | 0 | 0 |
|  | NAcbSh | 0 | 0 | 0 | 0 | 0 | 0 | 0 |
|  | PRL | 762 | 528 | 339 | 180 | 135 | 330 | 2274 |
|  | IL | 0 | 0 | 0 | 0 | 0 | 0 | 0 |
|  | Total | 762 | 528 | 339 | 180 | 135 | 330 | 2274 |
| 94_03 | NAcbC | 1050 | 1188 | 918 | 720 | 168 | 474 | 4518 |
|  | NAcbSh | 1050 | 1188 | 918 | 720 | 168 | 474 | 4518 |
|  | PRL | 875 | 990 | 765 | 600 | 140 | 395 | 3765 |
|  | IL | 350 | 396 | 306 | 240 | 56 | 158 | 1506 |
|  | Total | 3325 | 3762 | 2907 | 2280 | 532 | 1501 | 14307 |
| 97_14 | NAcbC | 784 | 490 | 406 | 203 | 21 | 35 | 1939 |
|  | NAcbSh | 224 | 140 | 116 | 58 | 6 | 10 | 554 |
|  | PRL | 448 | 280 | 232 | 116 | 12 | 20 | 1108 |
|  | IL | 336 | 210 | 174 | 87 | 9 | 15 | 831 |
|  | Total | 1792 | 1120 | 928 | 464 | 48 | 80 | 4432 |
| 97_16 | NAcbC | 891 | 324 | 252 | 180 | 27 | 81 | 1755 |
|  | NAcbSh | 198 | 72 | 56 | 40 | 6 | 18 | 390 |
|  | PRL | 396 | 144 | 112 | 80 | 12 | 36 | 780 |
|  | IL | 0 | 0 | 0 | 0 | 0 | 0 | 0 |
|  | Total | 1485 | 540 | 420 | 300 | 45 | 135 | 2925 |
| Total | NAcbC | 11468 | 9422 | 5718 | 4195 | 1694 | 4269 | 36766 |
|  | NAcbSh | 8462 | 6369 | 3984 | 2993 | 920 | 2675 | 25403 |
|  | PRL | 11757 | 8905 | 5503 | 3839 | 1661 | 4037 | 35702 |
|  | IL | 6267 | 4801 | 2900 | 1975 | 919 | 2065 | 18927 |
|  | Grand Total | 37954 | 29497 | 18105 | 13002 | 5194 | 13046 | 116798 |

## *Supporting Table 5: Wait-start gamma60 power [-0.25 0.25]*

| **Model Term** | **χ^2^** | **Df** | **P** |
| --- | --- | --- | --- |
| Outcome | 36.7740 | 2 | 0.0000 |
| Reward history | 79.5526 | 1 | 0.0000 |
| Structure | 3.7307 | 3 | 1.0000 |
| Outcome x reward history | 22.5851 | 2 | 0.0001 |
| Outcome x brain structure | 5.3168 | 6 | 1.0000 |
| Reward history x brain structure | 84.8624 | 3 | 0.0000 |
| Outcome x reward history x brain structure | 25.1164 | 6 | 0.0029 |

## *Supporting Table 6: Wait-start theta power [0.75 2]*

| **Model Term** | **χ^2^** | **Df** | **P** |
| --- | --- | --- | --- |
| Outcome | 141.9753 | 2 | 0.0000 |
| Reward history | 1733.3557 | 1 | 0.0000 |
| Structure | 1.9361 | 3 | 1.0000 |
| Outcome x reward history | 37.4176 | 2 | 0.0000 |
| Outcome x brain structure | 8.9135 | 6 | 1.0000 |
| Reward history x brain structure | 305.9292 | 3 | 0.0000 |
| Outcome x reward history x brain structure | 9.3813 | 6 | 1.0000 |

## *Supporting Table 7: Nose-poke gamma60 power [-0.25 0]*

| **Model Term** | **χ^2^** | **Df** | **P** |
| --- | --- | --- | --- |
| Outcome | 118.6464 | 2 | 0.0000 |
| Reward history | 14.9085 | 1 | 0.0010 |
| Structure | 22.0294 | 3 | 0.0006 |
| Outcome x reward history | 5.3190 | 2 | 0.6298 |
| Outcome x brain structure | 24.7868 | 6 | 0.0034 |
| Reward history x brain structure | 8.6642 | 3 | 0.3070 |
| Outcome x reward history x brain structure | 17.2048 | 6 | 0.0770 |

## *Supporting Table 8: Nose-poke gamma60 power [0.25 2]*

| **Model Term** | **χ^2^** | **Df** | **P** |
| --- | --- | --- | --- |
| Outcome | 3808.9190 | 2 | 0.0000 |
| Reward history | 28.4831 | 1 | 0.0000 |
| Structure | 19.8324 | 3 | 0.0017 |
| Outcome x reward history | 103.6013 | 2 | 0.0000 |
| Outcome x brain structure | 131.6762 | 6 | 0.0000 |
| Reward history x brain structure | 3.0565 | 3 | 1.0000 |
| Outcome x reward history x brain structure | 5.3042 | 6 | 1.0000 |

## *Supporting Table 9: Nose-poke theta power [-1 0]*

| **Model Term** | **χ^2^** | **Df** | **P** |
| --- | --- | --- | --- |
| Outcome | 138.0494 | 2 | 0.0000 |
| Reward history | 396.8619 | 1 | 0.0000 |
| Structure | 1.1704 | 3 | 1.0000 |
| Outcome x reward history | 61.2473 | 2 | 0.0000 |
| Outcome x brain structure | 20.1705 | 6 | 0.0232 |
| Reward history x brain structure | 76.7073 | 3 | 0.0000 |
| Outcome x reward history x brain structure | 18.7017 | 6 | 0.0423 |

## *Supporting Table 10: Nose-poke theta power [0.25 0.9]*

| **Model Term** | **χ^2^** | **Df** | **P** |
| --- | --- | --- | --- |
| Outcome | 2180.3551 | 2 | 0.0000 |
| Reward history | 95.3585 | 1 | 0.0000 |
| Structure | 0.8591 | 3 | 1.0000 |
| Outcome x reward history | 20.6807 | 2 | 0.0003 |
| Outcome x brain structure | 29.0431 | 6 | 0.0005 |
| Reward history x brain structure | 15.1006 | 3 | 0.0156 |
| Outcome x reward history x brain structure | 3.8810 | 6 | 1.0000 |

## *Supporting Table 11: Nose-poke theta power [1 2]*

| **Model Term** | **χ^2^** | **Df** | **P** |
| --- | --- | --- | --- |
| Outcome | 5003.1587 | 2 | 0.0000 |
| Reward history | 77.6354 | 1 | 0.0000 |
| Structure | 1.2259 | 3 | 1.0000 |
| Outcome x reward history | 12.3085 | 2 | 0.0191 |
| Outcome x brain structure | 75.6191 | 6 | 0.0000 |
| Reward history x brain structure | 23.2824 | 3 | 0.0003 |
| Outcome x reward history x brain structure | 12.9701 | 6 | 0.3916 |

## *Supporting Table 12: Wait-start gamma60-delta PAC [-1 0]*

| **Model Term** | **χ^2^** | **Df** | **P** |
| --- | --- | --- | --- |
| Outcome | 31.1163 | 2 | 0.0000 |
| Reward history | 98.6007 | 1 | 0.0000 |
| Structure | 26.1907 | 3 | 0.0001 |
| Outcome x reward history | 24.4524 | 2 | 0.0000 |
| Outcome x brain structure | 12.3727 | 6 | 0.4874 |
| Reward history x brain structure | 3.0080 | 3 | 1.0000 |
| Outcome x reward history x brain structure | 9.5999 | 6 | 1.0000 |

## *Supporting Table 13: Nose-poke PAC [0.75 2]*

| **Model Term** | **χ^2^** | **Df** | **P** |
| --- | --- | --- | --- |
| Outcome | 610.6286 | 2 | 0.0000 |
| Reward history | 6.9690 | 1 | 0.0746 |
| Structure | 9.0884 | 3 | 0.2533 |
| Outcome x reward history | 11.1823 | 2 | 0.0336 |
| Outcome x brain structure | 18.9431 | 6 | 0.0383 |
| Reward history x brain structure | 10.5923 | 3 | 0.1273 |
| Outcome x reward history x brain structure | 6.4350 | 6 | 1.000 |

## *Supporting Table 14: Impulsivity Screening Scores*

| **Rat ID** | **SITI1** | **SITI2** | **LITI1** | **SITI3** | **SITI4** | **SITI5** | **SITI6** | **LITI2** | **SITI7** | **SITI8** | **SITI9** | **SITI10** | **LITI3** | **Average LITI** | **Impulsivity Group** |
| --- | --- | --- | --- | --- | --- | --- | --- | --- | --- | --- | --- | --- | --- | --- | --- |
| **101_44** | *10* | *12* | *72* | *4* | *17* | *19* | *20* | *57* | *5* | *19* | *34* | *19* | *78* | *69* | *HI* |
| **102_54** | *27* | *16* | *90* | *10* | *7* | *19* | *26* | *64* | *7* | *7* | *16* | *16* | *67* | *73.66* | *HI* |
| **158_02** | *7* | *11* | *67* | *4* | *7* | *14* | *16* | *139* | *1* | *1* | *5* | *5* | *51* | *85.66* | *HI* |
| **158_08** | *16* | *19* | *102* | *9* | *4* | *12* | *12* | *96* | *11* | *9* | *18* | *17* | *96* | *98* | *HI* |
| **158_30** | *4* | *0* | *47* | *4* | *4* | *19* | *19* | *135* | *6* | *9* | *12* | *10* | *48* | *76.66* | *Non-HI* |
| **158_34** | *4* | *3* | *30* | *1* | *0* | *2* | *5* | *47* | *0* | *2* | *2* | *1* | *41* | *39.33* | *Non-HI* |
| **158_40** | *7* | *8* | *35* | *4* | *4* | *10* | *6* | *51* | *2* | *13* | *3* | *8* | *26* | *37.33* | *Non-HI* |
| **158_47** | *14* | *11* | *95* | *0* | *21* | *14* | *19* | *70* | *13* | *23* | *20* | *37* | *91* | *85.33* | *HI* |
| **229_11** | *16* | *26* | *88* | *11* | *11* | *15* | *22* | *96* | *9* | *6* | *12* | *14* | *45* | *76.33* | *HI* |
| **229_17** | *19* | *15* | *75* | *20* | *13* | *12* | *18* | *73* | *19* | *31* | *47* | *26* | *95* | *81* | *HI* |
| **229_22** | *15* | *20* | *125* | *7* | *5* | *13* | *20* | *67* | *7* | *8* | *14* | *12* | *59* | *83.66* | *HI* |
| **229_23** | *24* | *28* | *92* | *8* | *10* | *10* | *18* | *68* | *13* | *12* | *12* | *7* | *90* | *83.33* | *HI* |
| **229_30** | *31* | *45* | *149* | *22* | *25* | *20* | *24* | *74* | *15* | *16* | *31* | *26* | *55* | *92.66* | *HI* |
| **229_45** | *37* | *25* | *90* | *16* | *8* | *17* | *21* | *103* | *10* | *13* | *23* | *21* | *47* | *80* | *Non-HI* |
| **94_03** | *9* | *1* | *86* | *5* | *2* | *30* | *8* | *73* | *2* | *2* | *5* | *1* | *75* | *78* | *HI* |
| **97_14** | *4* | *5* | *55* | *3* | *4* | *11* | *1* | *43* | *3* | *5* | *5* | *4* | *19* | *39* | *Non-HI* |
| **97_16** | *32* | *19* | *92* |  | *15* | *13* | *7* | *48* | *4* | *8* | *12* | *11* | *40* | *60* | *Non-HI* |

## *Supporting Table 15: LFP variables*

| **Variable Name** | **Description** |
| --- | --- |
| wG60NAcbC | In the window [-0.25 0.25] around wait-start we found a significant interaction between upcoming trial outcome, previous trial reward history and brain structure on gamma60 power (χ^2­^_6_ = 25.1164, p = 0.0029, supporting table 5). Based on the plot in Figure S2A, this variable takes the average difference in gamma60 power of LFPs recorded in NAcbC between previously rewarded and non-rewarded premature trials. |
| wG60PRL | As wG60NAcbC, using LFPs from PRL |
| wTNAcbC | In the window [0.75 2] around wait-start we found a significant interaction between previous trial reward history and brain structure on theta power (χ^2­^_3_ = 305.9292, p < 0.0001, supporting table 6). Therefore, this variable takes the average difference in theta power of LFPs recorded in NAcbC between previously rewarded and non-rewarded trials (averaging over all upcoming outcomes). |
| wTPRL | As wTNAcbC, using LFPs from PRL |
| pG60NAcbC | In the window [0.25 2] following nose-poking we found a significant interaction between trial outcome and brain structure on gamma60 power (χ^2­^_6_ = 131.6762, p < 0.0001, supporting table 8). This variable takes the average difference in gamma60 power between correct and error (incorrect or premature) responses for LFPs recorded in NAcbC. |
| pG60PRL | As pG60NAcbC, using LFPs from PRL |
| pTNAcbC | In the window [0.25 0.9] following nose-poking we found a significant interaction between trial outcome and brain structure on theta power (χ^2­^_6_ = 29.0431, p = 0.0005, supporting table 10). This variable takes the average difference in theta power between correct and error (incorrect or premature) responses for LFPs recorded in NAcbC. |
| pTPRL | As pTNAcbC, using LFPs from PRL |
| pPACNAcbC | In the window [0.75 2] following nose-poking we found a significant interaction between trial outcome and brain structure on gamma60-delta PAC (χ^2­^_6_ = 18.9431, p = 0.0383, supporting table 13). This variable takes the average difference in gamma60-delta PAC between correct and error (incorrect or premature) responses for LFPs recorded in NAcbC. |
| pPACPRL | As pPACNAcbC, using LFPs from PRL |

## *Supporting Table 16: PC loadings on LFP variables*

| **LFP Variable** | **PC1** | **PC2** | **PC3** | **PC4** |
| --- | --- | --- | --- | --- |
| wG60NAcbC | -0.03684 | 0.91476 | 0.05861 | -0.12909 |
| wG60PRL | 0.09823 | 0.70408 | -0.62206 | 0.14700 |
| wTNAcbC | -0.90165 | 0.13562 | 0.01176 | 0.17677 |
| wTPRL | -0.00006 | -0.39472 | -0.58387 | 0.52390 |
| pG60NAcbC | 0.70003 | -0.28630 | 0.00622 | -0.34964 |
| pG60PRL | 0.85204 | 0.32909 | 0.09995 | 0.23451 |
| pTNAcbC | -0.33615 | 0.19956 | 0.12564 | 0.81550 |
| pTPRL | 0.07246 | -0.14462 | -0.05008 | 0.98831 |
| pPACNAcbC | 0.07098 | -0.07941 | 0.96241 | 0.06983 |
| pPACPRL | 0.42804 | 0.37204 | 0.50726 | 0.04594 |

## *Supporting Table 17: Factor scores for all rats*

| **Rat ID** | **PC1** | **PC2** | **PC3** | **PC4** | **screening** | **Average recording session %premature** | **group** |
| --- | --- | --- | --- | --- | --- | --- | --- |
| 101_44 | -0.4194 | -0.2732 | -1.2952 | 1.6721 | 69.00 | 10.02 | HI |
| 102_54 | -1.0222 | -0.8771 | 1.2273 | -0.304 | 73.67 | 11.22 | HI |
| 158_02 | 1.139 | 1.359 | 0.8367 | 0.8354 | 85.67 | 57.34 | HI |
| 158_08 | 1.8384 | -0.9434 | 0.1468 | 1.1699 | 98.00 | 3.92 | HI |
| 158_30 | -0.5277 | 0.8672 | -0.0245 | -0.3185 | 76.67 | 27.98 | Non-HI |
| 158_34 | -0.2962 | 0.8529 | -0.0665 | -0.3993 | 39.33 | 29.89 | Non-HI |
| 158_47 | 0.6901 | -1.1978 | 1.3576 | -0.416 | 85.33 | 19.63 | HI |
| 229_11 | -0.7804 | 0.965 | -1.5631 | -0.4618 | 76.33 | 12.63 | HI |
| 229_17 | -0.1155 | 1.188 | -0.8152 | -0.7037 | 81.00 | 33.93 | HI |
| 229_22 | 0.5083 | -0.5238 | 0.1665 | -1.1824 | 83.67 | 9.31 | HI |
| 229_23 | -0.7408 | 1.1402 | 0.2321 | -0.1823 | 83.33 | 17.59 | HI |
| 229_30 | 0.3441 | -1.0174 | -0.8605 | -1.3628 | 92.67 | 11.42 | HI |
| 94_03 | -1.4415 | 0.1804 | 0.5671 | 0.9355 | 78.00 | 15.65 | HI |
| 97_14 | -0.0942 | 0.2965 | 0.0594 | 0.0176 | 39.00 | 3.01 | Non-HI |
| 97_16 | -0.4299 | 0.0667 | 0.2238 | -0.8701 | 60.00 | 6.03 | Non-HI |
